# Intake of individual fatty acids and risk of prostate cancer in the European prospective investigation into cancer and nutrition

**DOI:** 10.1002/ijc.32233

**Published:** 2019-03-14

**Authors:** Aurora Perez‐Cornago, Inge Huybrechts, Paul N. Appleby, Julie A. Schmidt, Francesca L. Crowe, Kim Overvad, Anne Tjønneland, Tilman Kühn, Verena Katzke, Antonia Trichopoulou, Anna Karakatsani, Eleni Peppa, Sara Grioni, Domenico Palli, Carlotta Sacerdote, Rosario Tumino, H. Bas Bueno‐de‐Mesquita, Nerea Larrañaga, Maria‐Jose Sánchez, J. Ramón Quirós, Eva Ardanaz, María‐Dolores Chirlaque, Antonio Agudo, Anders Bjartell, Peter Wallström, Veronique Chajes, Konstantinos K. Tsilidis, Dagfinn Aune, Elio Riboli, Ruth C. Travis, Timothy J. Key

**Affiliations:** ^1^ Cancer Epidemiology Unit, Nuffield Department of Population Health University of Oxford Oxford United Kingdom; ^2^ Dietary Exposure Assessment Group International Agency for Research on Cancer Lyon France; ^3^ Institute of Applied Health Research, College of Medical and Dental Sciences University of Birmingham Birmingham United Kingdom; ^4^ Department of Public Health, Section for Epidemiology Aarhus University Aarhus C Denmark; ^5^ Danish Cancer Society Research Center Copenhagen Denmark; ^6^ Division of Cancer Epidemiology German Cancer Research Center (DKFZ) Heidelberg Germany; ^7^ Hellenic Health Foundation Athens Greece; ^8^ Department of Pulmonary Medicine, School of Medicine National and Kapodistrian University of Athens, “ATTIKON” University Hospital Haidari Greece; ^9^ Epidemiology and Prevention Unit Fondazione IRCCS Istituto Nazionale dei Tumori Milan Italy; ^10^ Cancer Risk Factors and Life‐Style Epidemiology Unit Institute for Cancer Research, Prevention and Clinical Network – ISPRO Florence Italy; ^11^ Unit of Cancer Epidemiology Città della Salute e della Scienza University‐Hospital and Center for Cancer Prevention (CPO) Turin Italy; ^12^ Department of Cancer Registry and Histopathology "Civic ‐ M.P. Arezzo" Hospital ASP Ragusa Italy; ^13^ Department for Determinants of Chronic Diseases (DCD) National Institute for Public Health and the Environment (RIVM) Bilthoven The Netherlands; ^14^ Department of Gastroenterology and Hepatology University Medical Centre Utrecht The Netherlands; ^15^ Department of Epidemiology and Biostatistics, The School of Public Health Imperial College London London United Kingdom; ^16^ Department of Social and Preventive Medicine, Faculty of Medicine University of Malaya Kuala Lumpur Malaysia; ^17^ Department of Basque Regional Health Public Health Division of Gipuzkoa‐BIODONOSTIA Guipúzcoa Spain; ^18^ CIBER of Epidemiology and Public Health Madrid Spain; ^19^ Escuela Andaluza de Salud Pública, Instituto de Investigación Biosanitaria ibs.GRANADA Hospitales Universitarios de Granada/Universidad de Granada Granada Spain; ^20^ Public Health Directorate Asturias Spain; ^21^ Navarra Public Health Institute Pamplona Spain; ^22^ IdiSNA, Navarra Institute for Health Research Pamplona Spain; ^23^ Department of Epidemiology, Regional Health Council Murcia Spain; ^24^ Department of Health and Social Sciences Universidad de Murcia Murcia Spain; ^25^ Unit of Nutrition and Cancer, Cancer Epidemiology Research Program Catalan Institute of Oncology‐IDIBELL. L'Hospitalet de Llobregat Barcelona Spain; ^26^ Department of Translational Medicine, Medical Faculty Lund University Malmö Sweden; ^27^ Department of Urology Skåne University Hospital Malmö Sweden; ^28^ Nutrition Epidemiology Research Group, Department of Clinical Sciences Lund University Malmö Sweden; ^29^ Clinical Research Centre Malmö University Hospital Malmö Sweden; ^30^ Department of Nutrition and Metabolism International Agency for Research on Cancer Lyon France; ^31^ Department of Hygiene and Epidemiology University of Ioannina, School of Medicine Ioannina Greece; ^32^ Department of Epidemiology and Biostatistics, School of Public Health Imperial College London London United Kingdom; ^33^ Department of Nutrition Bjørknes University College Oslo Norway; ^34^ Department of Endocrinology, Morbid Obesity and Preventive Medicine Oslo University Hospital Oslo Norway

**Keywords:** prostate cancer, individual fatty acids, tumor subtypes, prospective

## Abstract

The associations of individual dietary fatty acids with prostate cancer risk have not been examined comprehensively. We examined the prospective association of individual dietary fatty acids with prostate cancer risk overall, by tumor subtypes, and prostate cancer death. 142,239 men from the European Prospective Investigation into Cancer and Nutrition who were free from cancer at recruitment were included. Dietary intakes of individual fatty acids were estimated using center‐specific validated dietary questionnaires at baseline and calibrated with 24‐h recalls. Multivariable Cox regression models were used to estimate hazard ratios (HRs) and 95% confidence intervals (CIs). After an average follow‐up of 13.9 years, 7,036 prostate cancer cases and 936 prostate cancer deaths were ascertained. Intakes of individual fatty acids were not related to overall prostate cancer risk. There was evidence of heterogeneity in the association of some short chain saturated fatty acids with prostate cancer risk by tumor stage (*p*_heterogeneity_ < 0.015), with a positive association with risk of advanced stage disease for butyric acid (4:0; HR_1SD_ = 1.08; 95%CI = 1.01–1.15; *p*‐trend = 0.026). There were no associations with fatal prostate cancer, with the exception of a slightly higher risk for those who consumed more eicosenoic acid (22:1n‐9c; HR_1SD_ = 1.05; 1.00–1.11; *p*‐trend = 0.048) and eicosapentaenoic acid (20:5n‐3c; HR_1SD_ = 1.07; 1.00–1.14; *p*‐trend = 0.045). There was no evidence that dietary intakes of individual fatty acids were associated with overall prostate cancer risk. However, a higher intake of butyric acid might be associated with a higher risk of advanced, whereas intakes of eicosenoic and eicosapentaenoic acids might be positively associated with fatal prostate cancer risk.

## Abstract 2

**What's new?**

Are individual dietary fatty acids associated with prostate cancer development and progression? In this large, prospective study, the authors found that for prostate cancer overall, the answer is no. However, a higher intake of butyric acid may be associated with an increased risk of advanced‐stage prostate cancer, and higher intakes of eicosenoic and eicosapentaenoic acids may be positively associated with risk of lethal prostate cancer.

**Abbreviations**
BMI: body mass index
CIs: confidence intervals
EPIC: European Prospective Investigation into Cancer and Nutrition
FFQ: food‐frequency questionnaire
IGF‐I: insulin‐like growth factor‐I
ICD: International Statistical Classification of Diseases, Injuries and Causes of Death
HRs: hazard ratios
MUFAs: monounsaturated fatty acids
NNDSR = USDA: National Nutrient Database for Standard Reference of the United States
PIN: prostatic intraepithelial neoplasia
PSA: prostate‐specific antigen
PUFAs: polyunsaturated fatty acids
SFAs: saturated fatty acids
SDs: standard deviations
TNM: tumor‐node‐metastasis
UK: United Kingdom
WCRF/AICR: World Cancer Research Fund/American Institute for Cancer Research

## Introduction

Prostate cancer is the most frequently diagnosed cancer in men in Europe,^1^ but the well‐established risk factors age, ethnicity, genetic factors and family history of the disease are not modifiable.^2^, ^3^ There is also evidence that circulating insulin‐like growth factor‐I (IGF‐I) is related to higher overall prostate cancer risk,^4^ and obesity has been associated with a higher risk of aggressive disease.^5^ Moreover, the wide international variation in prostate cancer incidence and the changing rates observed in migrant studies suggest that environmental and lifestyle factors, such as dietary factors, are possible risk factors for the disease.^6^ However, some of this international variation is due to differences between countries in prostate‐specific antigen (PSA) testing, which has especially increased the diagnosis of nonaggressive tumors^1^; therefore, to provide more clarity on prostate cancer etiology, it is important that analytical studies characterize prostate cancer by stage, grade and fatality of the disease.

The possible role of total and specific types of dietary fats in relation to prostate cancer development and progression has attracted much interest.^7^, ^8^ The latest meta‐analysis from the World Cancer Research Fund/American Institute for Cancer Research (WCRF/AICR) stated that the evidence was limited and no conclusion could be reached on whether consumption of total fat, saturated fatty acids (SFAs), monounsaturated fatty acids (MUFAs), or polyunsaturated fatty acids (PUFAs) is associated with overall prostate cancer risk or with risk for ‘advanced/high grade’ prostate cancer.^2^, ^9^ However, this meta‐analysis did not differentiate between stage and grade of the disease because of the small number of available studies with data on both these outcomes, and associations with prostate cancer death were not available. Moreover, recent studies have shown that individual fatty acids may confer heterogeneous health effects,^10^, ^11^, ^12^ which might explain the current inconclusive results on the role of dietary fat on prostate cancer.

The aim of this study was to examine the association of intakes of individual dietary fatty acids with the risk of prostate cancer, and to examine whether any associations differ by tumor grade, stage, or for death from prostate cancer in the European Prospective Investigation into Cancer and Nutrition (EPIC).

## Material and Methods

### Subjects and study design

EPIC includes 153,457 men recruited between 1992 and 2000 from 19 centers, most aged 35–70 years, in 19 centers in eight European countries (Denmark, Germany, Greece, Italy, Netherlands, Spain, Sweden and United Kingdom (UK)) (more details in Supporting Information Methods). The details of the study design used in the EPIC study have been described elsewhere.^13^ Men were not eligible for this analysis if they were diagnosed with cancer (except nonmelanoma skin cancer) before recruitment (*n* = 3,972), if they had missing dates of prostate cancer diagnosis (*n* = 14) or follow‐up (*n* = 1,433), or if they were aged <20 years at recruitment (*n* = 2). Men were also excluded if they had no nondietary or dietary data, or if they had an extreme energy intake in relation to estimated requirement (top and bottom 1%, *n* = 5,766).^14^ Complete data on diet and follow‐up for prostate cancer were available for 142,239 men (Supporting Information Fig. S1).

### Assessment of dietary intake and other predictor variables

At baseline, information was collected on lifestyle, health status, socio‐demographic characteristics, anthropometry and medical history.^13^ Dietary intake during the year before enrolment was measured by country‐ or center‐specific validated food frequency questionnaires (FFQs) or diet histories, as previously described.^13^, ^15^ To correct for any systematic under‐ or overestimation of dietary intake across the participating centers, dietary intakes from the questionnaires were calibrated using a standardized, computer‐based, 24‐h dietary recall method in an 8% random sample of the whole EPIC cohort.

In order to estimate the intakes of individual fatty acids, the EPIC Nutrient Database (ENDB) was matched with the National Nutrient Database for Standard Reference of the United States (NNDSR; developed at the USDA).^15^, ^16^ The fatty acid intakes reported in this manuscript were obtained through this extra USDA matching (more details in Supporting Information Methods). Due to the very small amounts of some individual fatty acids, we only included those with a mean total intake of at least 0.05 g/day in these analyses, with the exception of docosapentaenoic acid (22:5n‐3c), which was included due to its previously suggested role in prostate cancer risk.^12^ Fatty acids were presented and analyzed as grams per 1,000 kcal/day in order to control for confounding by total energy intake.^17^ Short chain fatty acids were strongly correlated with each other (Supporting Information Table S2A), probably because of their shared food sources^18^; therefore, in addition to analyzing short chain SFAs individually, they were also combined together in groups as 4:0–10:0 and 12:0–14:0.^10^

### Ascertainment of prostate cancer

The main source of information on cancer incidence, tumor subtypes and vital status was population‐based cancer and mortality registries. In Germany and Greece follow‐up was based on a combination of methods, including health insurance records, cancer and pathology registries, as well as active follow‐up through participants or relatives; self‐reported incident cancers were verified through medical records. Follow‐up began at the date of recruitment and was censored at the date of last known contact, or at the date of diagnosis of cancer, death, emigration or the end of the follow‐up period, whichever came first. Prostate cancer (*n* = 7,036) was defined as code C61 in the 10th Revision of the International Statistical Classification of Diseases, Injuries and Causes of Death (ICD).^19^

Grade (based on Gleason sum) was classified as low‐intermediate (Gleason sum of <8, or grade coded as well, moderately, or poorly differentiated; *n* = 3,757) or high (Gleason sum of ≥8, or grade coded as undifferentiated; *n* = 726) grade. Information on stage was based on tumor‐node‐metastasis (TNM) staging code. Localized stage included those confined within the prostate and with no metastases at diagnosis (TNM staging score of ≤T_2_ and N_0_/N_x_ and M_0_, or stage coded in the recruitment center as localized; *n* = 2,641). Advanced cases included tumors that had spread beyond the prostate at diagnosis (T_3_‐T_4_ and/or N_1_‐N_3_ and/or M_1_, and/or stage coded in the recruitment center as metastatic; *n* = 1,389). Fatal cases were those who died of prostate cancer (*n* = 936).

### Statistical analysis

Baseline characteristics of the study population were calculated across fifths of total SFAs, MUFAs and PUFAs intake in grams/1000 kcal and presented as means with standard deviations (SDs) for continuous variables or percentages for categorical variables. Pearson correlations between intakes of individual SFAs, MUFAs and PUFAs were calculated.

Each individual fatty acid was divided into fifths of intake in grams/1000 kcal/day based on the distribution in the EPIC cohort and also modeled as continuous variables per SD higher intake.

Hazard ratios (HRs) and 95% confidence intervals (CIs) were estimated using Cox proportional hazards models using attained age as the underlying time variable. The date of last follow‐up ranged from January 2011 in Germany to October 2013 in Spain. All analyses were stratified by center and age (<50, 50–54.9, 55–59.9, 60–64.9, 65–69.9, and ≥70 years) at recruitment. To check for violation of the proportional hazards assumption we used time‐varying covariates and Schoenfeld residuals, which did not indicate violation from the proportional hazards assumption. Tests for linear trend were performed using continuous values for dietary fatty acids, with increments based on one SD increase. All models were adjusted for educational level (no degree or equivalent, degree or equivalent, unknown), smoking status (never, former, current, unknown), marital status (married or cohabiting, not married or cohabiting, unknown), diabetes (no, yes, unknown), physical activity (inactive, moderately inactive, moderately active, active, unknown^20^), height (<170, 170–174, 175–179, ≥180 cm, unknown), BMI (<22.5, 22.5–24.9, 25–29.9, ≥30 kg/m^2^, unknown), and total energy intake (fifths). Participants with missing values were assigned an “unknown” category; <3% of values were missing for each covariate, with the exception of marital status, for which 30% of values were missing.

Tests for heterogeneity of trends for histological grade (low‐intermediate or high), tumor stage (localized or advanced), and time between blood collection and diagnosis (<5 years, ≥5 years)] were performed. For this, we fitted separate models for each subgroup assuming independence of the HRs using a method analogous to competing risks, and compared the risk coefficients and standard errors in the subgroups of interest after excluding cases of unknown grade or stage.^21^

All analyses were performed using Stata version 14.1 (Stata Corporation, College Station, TX), all tests of significance were two‐sided, and a *p*‐value less than 0.05 was considered statistically significant. Conventional *p*‐values are shown but the results were interpreted in the light of the number of tests performed.

## Results

A total of 7,036 men were diagnosed with prostate cancer after an average follow‐up time of 13.9 years. The median age at prostate cancer diagnosis was 68 years (range, 41–95 years). Table 1 shows the characteristics of the study participants at baseline. Some baseline characteristics varied by fatty acids consumption. For example, men in the highest fifth of SFAs intake were more likely to have an education degree and have a higher total energy intake. Men in the highest fifths of MUFAs and PUFAs intake were more likely to be younger at recruitment and older at prostate cancer diagnosis.

**Table 1:** Baseline characteristics of 142,239 men in EPIC (1992–2013) according to observed SFAs, MUFAs and PUFAs intakes

	Fifths of observed SFAs intake			Fifths of observed MUFAs intake			Fifths of observed PUFAs intake		
	1	3	5	1	3	5	1	3	5
No. of men	28,448	28,448	28,447	28,448	28,448	28,447	28,448	28,448	28,447
Age at recruitment^1^, y	52.2 (9.7)	51.7 (10.3)	51.1 (10.3)	52.8 (10.6)	51.8 (9.8)	50.6 (10.5)	52.3 (8.5)	51.7 (10.1)	51.0 (11.4)
Age at diagnosis^1^, y	67.8 (6.4)	68.1 (6.8)	67.5 (6.9)	68.7 (6.8)	68.0 (6.5)	66.9 (6.7)	67.1 (6.2)	67.8 (6.6)	68.5 (7.0)
*Smoking, n (%)*									
Never	8,990 (31.6)	9,475 (33.3)	9,526 (33.5)	9,958 (35.0)	9,713 (34.1)	8,175 (28.7)	7,794 (27.4)	9,721 (34.2)	10,262 (36.1)
Former	10,650 (37.4)	10,266 (36.1)	9,838 (34.6)	11,130 (39.1)	10,299 (36.2)	9,400 (33.0)	10,983 (38.6)	10,481 (36.8)	9,347 (32.9)
Current	8,479 (29.8)	8,281 (29.1)	8,704 (30.6)	6,979 (24.5)	8,137 (28.6)	10,193 (35.8)	9,375 (33.0)	7,892 (27.7)	8,324 (29.3)
*Educational level, n (%)*									
No degree	20,722 (72.8)	20,560 (72.3)	18,919 (66.5)	18,064 (63.5)	19,806 (69.6)	22,702 (79.8)	21,226 (74.6)	19,770 (69.5)	19,954 (70.1)
Degree	6,638 (23.3)	7,156 (25.2)	8,918 (31.3)	8,724 (30.7)	7,996 (28.1)	5,552 (19.5)	6,887 (24.2)	8,014 (28.2)	7,139 (25.1)
*Physical activity, n (%)*									
Inactive	5,527 (19.4)	5,134 (18.0)	5,539 (19.5)	5,366 (18.9)	4,745 (16.7)	6,816 (24.0)	5,002 (17.6)	5,221 (18.4)	5,861 (20.6)
Moderately inactive	8,502 (29.9)	8,721 (30.7)	8,854 (31.1)	8,426 (29.6)	8,770 (30.8)	8,782 (30.9)	9,033 (31.8)	8,618 (30.3)	8,285 (29.1)
Moderately active	6,950 (24.4)	6,793 (23.9)	6,958 (24.5)	6,773 (23.8)	6,906 (24.3)	7,013 (24.7)	6,943 (24.4)	7,004 (24.6)	6,992 (24.6)
Active	7,190 (25.3)	7,088 (24.9)	6,320 (22.2)	7,297 (25.7)	7,271 (25.6)	5,570 (19.6)	7,272 (25.6)	6,870 (24.1)	6,516 (22.9)
*Diabetes at baseline, n (%)*									
No	26,578 (93.4)	26,848 (94.4)	26,869 (94.5)	26,618 (93.6)	26,937 (94.7)	26,610 (93.5)	27,048 (95.1)	26,715 (93.9)	26,680 (93.8)
Yes	1,203 (4.2)	923 (3.2)	1,064 (3.7)	1,024 (3.6)	864 (3.0)	1,459 (5.1)	981 (3.4)	1,104 (3.9)	961 (3.4)
*Marital status, n (%)*									
Married	11,222 (39.4)	16,441 (57.8)	19,193 (67.5)	14,234 (50.0)	15,587 (54.8)	18,378 (64.6)	16,023 (56.3)	15,925 (56.0)	15,389 (54.1)
Not married	2,944 (10.3)	3,624 (12.7)	4,983 (17.5)	4,210 (14.8)	3,707 (13.0)	3,117 (11.0)	3,084 (10.8)	3,785 (13.3)	4,395 (15.4)
Height^1^, cm	173.1 (7.4)	175.0 (7.3)	175.8 (7.1)	175.4 (7.0)	175.5 (7.2)	172.2 (7.5)	173.5 (7.3)	175.2 (7.2)	174.9 (7.5)
BMI^1^, kg/m^2^	26.8 (3.7)	26.4 (3.6)	26.3 (3.7)	26.3 (3.7)	26.2 (3.5)	27.3 (3.8)	26.7 (3.6)	26.5 (3.6)	26.2 (3.8)
Total energy intake^1^, Kcal/d	2,289 (641)	2,426 (650)	2,508 (701)	2,232 (623)	2,442 (641)	2,500 (703)	2,431 (679)	2,391 (648)	2,438 (676)

Percentages do not match due to missing data. Fifths calculated from g/1000 kcal of each fatty acid.

1 Values are means (SD).

Abbreviations: BMI, body mass index; MUFAs, monounsaturated fatty acids; PUFAs, polyunsaturated fatty acids; SFAs, saturated fatty acids.

Palmitic acid (16:0), oleic acid (18:1n‐9c) and linoleic acid (18:2n‐6c) were the largest contributors to total SFAs, MUFAs and PUFAs intake, respectively (Supporting Information Table S1). Butyric acid (4:0), caproic acid (6:0), caprylic acid (8:0) and capric acid (10:0) were strongly correlated with each other (correlation coefficients ranged from 0.840 to 0.972). There was also a strong correlation between palmitic acid (16:0) and stearic acid (18:0) (correlation coefficient 0.814, Supporting Information Table S2A). Although individual MUFAs were correlated, these correlations were not very strong (Supporting Information Table S2B). The PUFAs eicosapentaenoic acid (20:5n‐3c), docosapentaenoic acid (22:5n‐3c) and docosahexaenoic acid (22:6n‐3c) were also strongly correlated (correlation coefficients ranged from 0.872 to 0.968, Supporting Information Table S2C).

The associations of intakes of individual SFAs, MUFAs and PUFAs with risk for overall prostate cancer, prostate cancer subdivided by grade and stage of disease, and for prostate cancer death, using both the observed and calibrated intakes, are shown in Tables 2 (Supporting Information Table S3 for observed intakes), 3 (Supporting Information Table S4 for observed intakes), and 4 (Supporting Information Table S5 for observed intakes), respectively. Results for observed and calibrated intakes were similar in direction; therefore, from here on we will only report calibrated results.

**Table 2:** Multivariable‐adjusted hazard ratios (95% CI) for prostate cancer per 1‐SD increase of total fat and individual saturated fatty acids intake in 142,239 men in EPIC (1992–2013)

		Calibrated		
	No. cases	HR (95% CI)^1^	P trend^2^	P het^3^
*Total Fat*				
Total prostate cancer	7,036	0.99 (0.96–1.02)	0.389	
Grade				
Low	3,757	0.95 (0.91–1.00)	0.038	
High	726	1.04 (0.95–1.14)	0.419	0.084
Stage				
Localized	2,641	0.98 (0.93–1.04)	0.544	
Advanced	1,389	1.01 (0.95–1.08)	0.708	0.392
Fatal prostate cancer	936	1.00 (0.92–1.08)	0.965	
*Total SFAs*				
Total prostate cancer	7,036	1.00 (0.96–1.03)	0.850	
Grade				
Low	3,757	0.96 (0.92–1.01)	0.144	
High	726	1.07 (0.96–1.19)	0.234	0.041
Stage				
Localized	2,641	0.97 (0.91–1.02)	0.239	
Advanced	1,389	1.05 (0.97–1.13)	0.257	0.052
Fatal prostate cancer	936	1.02 (0.92–1.12)	0.723	
*Butyric acid (4:0)*				
Total prostate cancer	7,036	1.01 (0.98–1.04)	0.496	
Grade				
Low	3,757	0.99 (0.95–1.03)	0.724	
High	726	1.07 (0.97–1.17)	0.162	0.035
Stage				
Localized	2,641	0.96 (0.92–1.01)	0.135	
Advanced	1,389	1.08 (1.01–1.15)	0.026	0.004
Fatal prostate cancer	936	1.02 (0.94–1.12)	0.599	
*Caproic acid (6:0)*				
Total prostate cancer	7,036	1.00 (0.97–1.04)	0.819	
Grade				
Low	3,757	0.98 (0.94–1.02)	0.380	
High	726	1.06 (0.97–1.16)	0.198	0.034
Stage				
Localized	2,641	0.96 (0.91–1.00)	0.071	
Advanced	1,389	1.06 (1.00–1.13)	0.053	0.006
Fatal prostate cancer	936	1.03 (0.95–1.12)	0.476	
*Caprylic acid (8:0)*				
Total prostate cancer	7,036	1.00 (0.97–1.03)	0.937	
Grade				
Low	3,757	0.97 (0.93–1.01)	0.156	
High	726	1.04 (0.95–1.14)	0.394	0.068
Stage				
Localized	2,641	0.95 (0.91–1.00)	0.042	
Advanced	1,389	1.05 (0.99–1.12)	0.122	0.013
Fatal prostate cancer	936	1.05 (0.97–1.13)	0.272	
*Capric acid (10:0)*				
Total prostate cancer	7,036	1.01 (0.97–1.04)	0.775	
Grade				
Low	3,757	0.98 (0.94–1.03)	0.397	
High	726	1.06 (0.95–1.17)	0.317	0.072
Stage				
Localized	2,641	0.96 (0.90–1.01)	0.116	
Advanced	1,389	1.06 (0.99–1.14)	0.093	0.023
Fatal prostate cancer	936	1.04 (0.94–1.14)	0.481	
*Lauric acid (12:0)*				
Total prostate cancer	7,036	1.00 (0.97–1.04)	0.791	
Grade				
Low	3,757	0.98 (0.93–1.02)	0.333	
High	726	1.07 (0.97–1.19)	0.182	0.102
Stage				
Localized	2,641	0.96 (0.91–1.01)	0.153	
Advanced	1,389	1.07 (0.99–1.14)	0.079	0.078
Fatal prostate cancer	936	1.06 (0.96–1.17)	0.253	
*Myristic acid (14:0)*				
Total prostate cancer	7,036	1.00 (0.97–1.04)	0.916	
Grade				
Low	3,757	0.97 (0.93–1.02)	0.208	
High	726	1.06 (0.96–1.17)	0.265	0.028
Stage				
Localized	2,641	0.96 (0.91–1.01)	0.113	
Advanced	1,389	1.06 (0.98–1.14)	0.129	0.013
Fatal prostate cancer	936	1.04 (0.95–1.14)	0.426	
*Pentadecanoic acid (15:0)*				
Total prostate cancer	7,036	0.98 (0.94–1.01)	0.152	
Grade				
Low	3,757	0.99 (0.94–1.04)	0.618	
High	726	0.93 (0.84–1.03)	0.146	0.427
Stage				
Localized	2,641	0.96 (0.91–1.02)	0.212	
Advanced	1,389	0.97 (0.91–1.04)	0.430	0.511
Fatal prostate cancer	936	0.97 (0.89–1.05)	0.410	
*Palmitic acid (16:0)*				
Total prostate cancer	7,036	0.99 (0.95–1.03)	0.627	
Grade				
Low	3,757	0.96 (0.91–1.01)	0.104	
High	726	1.06 (0.95–1.19)	0.282	0.064
Stage				
Localized	2,641	0.97 (0.91–1.03)	0.324	
Advanced	1,389	1.03 (0.95–1.12)	0.436	0.131
Fatal prostate cancer	936	1.02 (0.92–1.12)	0.709	
*Margaric acid (17:0)*				
Total prostate cancer	7,036	0.99 (0.96–1.02)	0.549	
Grade				
Low	3,757	0.98 (0.94–1.03)	0.442	
High	726	1.03 (0.93–1.13)	0.562	0.130
Stage				
Localized	2,641	0.96 (0.92–1.01)	0.141	
Advanced	1,389	1.06 (0.99–1.13)	0.099	0.036
Fatal prostate cancer	936	0.98 (0.90–1.08)	0.738	
*Stearic acid (18:0)*				
Total prostate cancer	7,036	0.99 (0.95–1.03)	0.628	
Grade				
Low	3,757	0.96 (0.90–1.01)	0.118	
High	726	1.06 (0.94–1.20)	0.349	0.087
Stage				
Localized	2,641	0.97 (0.90–1.04)	0.357	
Advanced	1,389	1.04 (0.95–1.14)	0.387	0.117
Fatal prostate cancer	936	0.99 (0.89–1.10)	0.799	
*Arachidic acid (20:0)*				
Total prostate cancer	7,036	0.99 (0.94–1.04)	0.801	
Grade				
Low	3,757	0.99 (0.93–1.06)	0.861	
High	726	0.95 (0.81–1.12)	0.565	0.963
Stage				
Localized	2,641	1.01 (0.94–1.10)	0.739	
Advanced	1,389	0.96 (0.86–1.07)	0.458	0.297
Fatal prostate cancer	936	0.89 (0.77–1.04)	0.140	
*Behenic acid (22:0)*				
Total prostate cancer	7,036	0.99 (0.95–1.03)	0.616	
Grade				
Low	3,757	0.99 (0.94–1.04)	0.707	
High	726	0.90 (0.76–1.06)	0.210	0.665
Stage				
Localized	2,641	1.02 (0.95–1.09)	0.581	
Advanced	1,389	0.94 (0.85–1.04)	0.243	0.275
Fatal prostate cancer	936	0.90 (0.77–1.04)	0.156	
*4:0–10:0*				
Total prostate cancer	7,036	1.01 (0.97–1.04)	0.718	
Grade				
Low	3,757	0.98 (0.94–1.03)	0.435	
High	726	1.06 (0.96–1.16)	0.234	0.043
Stage				
Localized	2,641	0.96 (0.91–1.01)	0.093	
Advanced	1,389	1.07 (1.00–1.14)	0.054	0.008
Fatal prostate cancer	936	1.03 (0.95–1.12)	0.464	
*12:0–14:0*				
Total prostate cancer	7,036	1.00 (0.97–1.04)	0.859	
Grade				
Low	3,757	0.97 (0.93–1.02)	0.229	
High	726	1.07 (0.96–1.18)	0.223	0.030
Stage				
Localized	2,641	0.96 (0.91–1.01)	0.124	
Advanced	1,389	1.06 (0.99–1.14)	0.103	0.016
Fatal prostate cancer	936	1.05 (0.96–1.16)	0.299	

Cox regression analysis. All models are stratified by centre and age at recruitment and adjusted for age (underlying time variable), educational level (no degree, degree or unknown), smoking status (never, former, current or unknown), marital status (married, not married, unknown), diabetes (yes, no, unknown), physical activity (inactive, moderately inactive, moderately active, active, unknown), height (<170, 170–174, 175–179, ≥180 cm or unknown), body mass index (<22.5, 22.5–24.9, 25–29.9, ≥30 kg/m^2^ or unknown), and total energy intake (fifths).

Low‐intermediate grade (Gleason score of <8, or grade coded as well, moderately, or poorly differentiated). High grade (Gleason score of ≥8, or grade coded as undifferentiated). Localized stage (TNM staging score of T0‐T2 and N0/Nx and M0, or stage coded in the recruitment centre as localized). Advanced stage (T3‐T4 and/or N1‐N3 and/or M1, and/or stage coded in the recruitment centre as metastatic).

1 HR (95% CI) estimated per 1‐SD increase in fatty acids intake.

2 Linear trends for HRs estimates over a continuous scale of the individual fatty acid.

3 *P*‐value from test for heterogeneity for the associations of intake the individual fatty acids with risk of prostate cancer categorized according to prostate tumor grade (low‐intermediate or high) and stage (localized or advanced).

Abbreviation: SFAs saturated fatty acids.

**Table 3:** Multivariable‐adjusted hazard ratios (95% CI) for prostate cancer per 1‐SD increase of individual monounsaturated fatty acids intake in 142,239 men in EPIC (1992–2013)

		Calibrated		
	No. cases	HR (95% CI)^1^	P trend^2^	P het^3^
*Total MUFAs*				
Total prostate cancer	7,036	0.98 (0.93–1.02)	0.303	
Grade				
Low	3,757	0.95 (0.90–1.01)	0.121	
High	726	1.04 (0.91–1.18)	0.613	0.411
Stage				
Localized	2,641	1.00 (0.93–1.08)	0.991	
Advanced	1,389	1.01 (0.91–1.11)	0.920	0.942
Fatal prostate cancer	936	0.98 (0.87–1.10)	0.676	
*Palmitoleic acid (16:1n‐7c)*				
Total prostate cancer	7,036	1.00 (0.94–1.05)	0.893	
Grade				
Low	3,757	0.96 (0.89–1.04)	0.353	
High	726	1.08 (0.91–1.28)	0.372	0.317
Stage				
Localized	2,641	1.01 (0.92–1.11)	0.779	
Advanced	1,389	1.02 (0.91–1.15)	0.742	0.731
Fatal prostate cancer	936	1.06 (0.91–1.23)	0.446	
*Oleic acid (18:1n‐9c)*				
Total prostate cancer	7,036	0.98 (0.94–1.03)	0.405	
Grade				
Low	3,757	0.96 (0.90–1.02)	0.182	
High	726	1.03 (0.89–1.18)	0.686	0.517
Stage				
Localized	2,641	0.99 (0.92–1.07)	0.847	
Advanced	1,389	1.02 (0.92–1.13)	0.777	0.945
Fatal prostate cancer	936	0.98 (0.87–1.11)	0.744	
*Eicosenoic acid (20:1n‐9c)*				
Total prostate cancer	7,036	1.01 (0.98–1.03)	0.540	
Grade				
Low	3,757	0.98 (0.94–1.02)	0.283	
High	726	1.05 (0.99–1.12)	0.094	0.174
Stage				
Localized	2,641	1.00 (0.95–1.04)	0.932	
Advanced	1,389	1.02 (0.97–1.06)	0.512	0.550
Fatal prostate cancer	936	1.05 (1.00–1.11)	0.048	
*Erucic acid (22:1n‐9c)*				
Total prostate cancer	7,036	1.01 (0.98–1.03)	0.500	
Grade				
Low	3,757	0.97 (0.93–1.02)	0.262	
High	726	1.06 (0.99–1.12)	0.091	0.129
Stage				
Localized	2,641	1.00 (0.95–1.04)	0.865	
Advanced	1,389	1.02 (0.98–1.07)	0.363	0.313
Fatal prostate cancer	936	1.05 (1.00–1.11)	0.055	

Cox regression analysis. All models are stratified by centre and age at recruitment and adjusted for age (underlying time variable), educational level (no degree, degree or unknown), smoking status (never, former, current or unknown), marital status (married, not married, unknown), diabetes (yes, no, unknown), physical activity (inactive, moderately inactive, moderately active, active, unknown), height (<170, 170–174, 175–179, ≥180 cm or unknown), body mass index (<22.5, 22.5–24.9, 25–29.9, ≥30 kg/m^2^ or unknown), and total energy intake (fifths).

Low‐intermediate grade (Gleason score of <8, or grade coded as well, moderately, or poorly differentiated). High grade (Gleason score of ≥8, or grade coded as undifferentiated). Localized stage (TNM staging score of T0‐T2 and N0/Nx and M0, or stage coded in the recruitment centre as localized). Advanced stage (T3‐T4 and/or N1‐N3 and/or M1, and/or stage coded in the recruitment centre as metastatic).

1 HR (95% CI) estimated per 1‐SD increase in fatty acids intake.

2 Linear trends for HRs estimates over a continuous scale of the individual fatty acid.

3 *p*‐value from test for heterogeneity for the associations of intake the individual fatty acids with risk of prostate cancer categorized according to prostate tumor grade (low‐intermediate or high) and stage (localized or advanced).

Abbreviations: MUFAs, monounsaturated fatty acids.

**Table 4:** Multivariable‐adjusted hazard ratios (95% CI) for prostate cancer per 1‐SD increase of individual polyunsaturated fatty acids intake in 142,239 men in EPIC (1992–2013)

		Calibrated		
	No. cases	HR (95% CI)^1^	P trend^2^	P het^3^
*Total PUFAs*				
Total prostate cancer	7,036	0.98 (0.95–1.02)	0.340	
Grade				
Low	3,757	0.96 (0.91–1.01)	0.086	
High	726	1.00 (0.91–1.11)	0.925	0.508
Stage				
Localized	2,641	0.99 (0.94–1.05)	0.830	
Advanced	1,389	0.97 (0.90–1.05)	0.434	0.777
Fatal prostate cancer	936	0.99 (0.90–1.09)	0.836	
*Linoleic acid (18:2n‐6c)*				
Total prostate cancer	7,036	0.98 (0.95–1.02)	0.311	
Grade				
Low	3,757	0.96 (0.91–1.00)	0.063	
High	726	1.01 (0.91–1.12)	0.857	0.450
Stage				
Localized	2,641	0.99 (0.93–1.05)	0.678	
Advanced	1,389	0.97 (0.90–1.05)	0.440	0.824
Fatal prostate cancer	936	0.99 (0.90–1.08)	0.822	
*α‐Linolenic acid (18:3n‐3c)*				
Total prostate cancer	7,036	0.99 (0.95–1.02)	0.502	
Grade				
Low	3,757	0.97 (0.92–1.02)	0.270	
High	726	1.01 (0.91–1.13)	0.817	0.514
Stage				
Localized	2,641	0.98 (0.92–1.05)	0.602	
Advanced	1,389	1.00 (0.93–1.08)	0.907	0.434
Fatal prostate cancer	936	0.99 (0.90–1.09)	0.839	
*Arachidonic acid (20:4n‐6c)*				
Total prostate cancer	7,036	0.99 (0.95–1.03)	0.638	
Grade				
Low	3,757	0.97 (0.92–1.03)	0.387	
High	726	0.94 (0.82–1.08)	0.381	0.404
Stage				
Localized	2,641	1.05 (0.97–1.13)	0.213	
Advanced	1,389	0.91 (0.82–1.01)	0.072	0.021
Fatal prostate cancer	936	1.03 (0.92–1.15)	0.636	
*Eicosapentaenoic acid (20:5n‐3c)*				
Total prostate cancer	7,036	1.02 (0.99–1.04)	0.272	
Grade				
Low	3,757	1.01 (0.96–1.05)	0.762	
High	726	0.99 (0.92–1.08)	0.895	0.660
Stage				
Localized	2,641	1.02 (0.98–1.07)	0.343	
Advanced	1,389	1.01 (0.95–1.07)	0.831	0.601
Fatal prostate cancer	936	1.07 (1.00–1.14)	0.045	
*Docosapentaenoic acid (22:5n‐3c)*				
Total prostate cancer	7,036	1.00 (0.97–1.03)	0.989	
Grade				
Low	3,757	1.00 (0.96–1.04)	0.809	
High	726	0.97 (0.90–1.05)	0.454	0.357
Stage				
Localized	2,641	1.03 (0.98–1.08)	0.251	
Advanced	1,389	1.00 (0.94–1.06)	0.914	0.229
Fatal prostate cancer	936	1.03 (0.97–1.10)	0.325	
*Docosahexaenoic acid (22:6n‐3c)*				
Total prostate cancer	7,036	1.01 (0.98–1.04)	0.504	
Grade				
Low	3,757	1.00 (0.96–1.05)	0.875	
High	726	0.97 (0.89–1.06)	0.523	0.388
Stage				
Localized	2,641	1.02 (0.97–1.07)	0.504	
Advanced	1,389	1.00 (0.94–1.07)	0.884	0.479
Fatal prostate cancer	936	1.06 (0.99–1.14)	0.083	

Cox regression analysis. All models are stratified by centre and age at recruitment and adjusted for age (underlying time variable), educational level (no degree, degree or unknown), smoking status (never, former, current or unknown), marital status (married, not married, unknown), diabetes (yes, no, unknown), physical activity (inactive, moderately inactive, moderately active, active, unknown), height (<170, 170–174, 175–179, ≥180 cm or unknown), body mass index (<22.5, 22.5–24.9, 25–29.9, ≥30 kg/m^2^ or unknown), and total energy intake (fifths).

Low‐intermediate grade (Gleason score of <8, or grade coded as well, moderately, or poorly differentiated). High grade (Gleason score of ≥8, or grade coded as undifferentiated). Localized stage (TNM staging score of T0‐T2 and N0/Nx and M0, or stage coded in the recruitment centre as localized). Advanced stage (T3‐T4 and/or N1‐N3 and/or M1, and/or stage coded in the recruitment centre as metastatic).

1 HR (95% CI) estimated per 1‐SD increase in fatty acids intake.

2 Linear trends for HRs estimates over a continuous scale of the individual fatty acid.

3 *P*‐value from test for heterogeneity for the associations of intake the individual fatty acids with risk of prostate cancer categorized according to prostate tumor grade (low‐intermediate or high) and stage (localized or advanced).

Abbreviation: PUFAs, polyunsaturated fatty acids.

Intakes of individual SFAs, MUFAs and PUFAs were not related to overall prostate cancer risk (Supporting Information Table S6 and Tables 2, 3 and 4). Removing BMI as a covariate in the model has no impact on any of the results. There was no evidence of heterogeneity in separate analyses by grade (Tables 2, 3 and 4 **)**. We found evidence of heterogeneity in the association of some SFAs [butyric acid (4:0), caproic acid (6:0), 4:0–10:0 combined and 12:0–14:0 combined] with prostate cancer risk by tumor stage (*p*_heterogeneity for all_ < 0.03; Table 2), with a positive association with risk of advanced stage disease for butyric acid (4:0; HR_1SD in calibrated intake_ = 1.08; 1.01–1.15; *p*‐trend = 0.026), and no significant associations with localized disease. There was also evidence of heterogeneity by tumor stage (*p*_heterogeneity_ = 0.021) in the association between arachidonic acid (20:4n‐6c) with prostate cancer risk, with a higher intake of this PUFA being weakly associated with a lower risk of advanced disease (HR_1SD in calibrated intake_ = 0.91, 0.82–1.01; *p*‐trend = 0.072; Table 4).

We observed no associations between intakes of individual dietary fatty acids and prostate cancer death, with the exception of a small increased risk for those with a higher intake of eicosenoic acid (22:1n‐9c; HR_1SD in calibrated intake_ = 1.05; 95% CI 1.00–1.11; *p*‐trend = 0.048; Table 3) and eicosapentaenoic acid (20:5n‐3c; HR_1SD in calibrated intake_ = 1.07; 95% CI 1.00–1.14; *p*‐trend = 0.045; Table 4).

Although there was some evidence of heterogeneity for the association of some long‐chain SFAs with total prostate cancer risk when subdivided by time between recruitment and diagnosis (<5 years, ≥5 years; Supporting Information Table S7), the associations at each of the follow‐up times were not statistically significant. There was no evidence of heterogeneity for the rest of individual fatty acids by follow‐up time.

## Discussion

In this large prospective study, intakes of individual fatty acids were not associated with overall prostate cancer risk. However, a higher intake of butyric acid was positively associated with risk for advanced stage prostate cancer. There was also a small increased risk of fatal prostate cancer risk with higher intakes of eicosenoic acid and eicosapentaenoic acid.

The possible association between fat intake (total fat and specific fatty acids) with prostate cancer risk and/or progression has generated considerable debate. Animal and cell studies have shown that dietary fat can promote metastasis.^22^, ^23^, ^24^ Several mechanisms that may underpin this association have been proposed. These include a positive association of total fat intake with IGF‐I^25^ and androgen^26^ concentrations and, based on experiments in mice, a possible role in the activation of the IGF‐Akt pathway and the proliferation of prostatic intraepithelial neoplasia (PIN) epithelial cells.^23^ Nevertheless, data from prospective studies are inconclusive.^2^ There are various differences between prospective studies that could account for the inconclusive findings, such as that most studies have combined grade and stage of the disease, and that the type of fatty acid rather than total amount may play a role in prostate cancer development and/or progression.

Our finding of an increased risk of advanced prostate cancer associated with higher butyric acid (4:0), caproic acid (6:0), and 4:0–10:0 combined intakes is difficult to put into context, as other prospective studies have not examined these associations. A previous survival analyses among Swedish men initially diagnosed with localized prostate cancer found that a higher intake of short chain fatty acids (4:0–10:0) may increase risk of prostate cancer death^27^; myristic acid (14:0) was also related with worse prostate cancer survival in this study, but we did not find an association of this SFA with prostate cancer risk. A prospective study in Japanese men also found a positive association between myristic and palmitic acid and prostate cancer risk.^28^ Dairy products are the main dietary source of short chain fatty acids,^18^ which might be involved in prostate cancer etiology.^2^ Therefore, it is also possible that the observed associations are driven by other compounds present in dairy products, such as protein or calcium.^29^ However, when we further adjusted the multivariable‐adjusted models for protein or calcium from dairy products, the significant association between butyric acid and advanced prostate cancer risk was maintained and was even a bit stronger (protein adjustment, HR_1SD in calibrated intake_ = 1.09; 1.02–1.16; *p*‐trend = 0.016; calcium adjustment, HR_1SD in calibrated intake_ = 1.09; 1.01–1.16; *p*‐trend = 0.020). Moreover, butter is particularly high in butyric acid and it is also a good source of phytanic acid, which has been related to prostate cancer in the Alpha‐Tocopherol, Beta‐Carotene Cancer Prevention Study,^30^ a cohort of Finnish male smokers. Phytanic acid is also high in fish, and as discussed below, we have also found a weak association between eicosapentaenoic acid and death from prostate cancer. However, circulating phytanic acid concentrations were not significantly associated with prostate cancer risk in the EPIC cohort.^31^

A higher intake of arachidonic acid (20:4n‐6c) was weakly associated with a lower risk of advanced disease, which is consistent with a previous individual participant meta‐analysis of prospective studies on circulating fatty acids that found an inverse association with aggressive prostate cancer.^12^ However, previous prospective studies have found no association between dietary arachidonic acid and prostate cancer risk.^32^, ^33^ The possible mechanisms whereby arachidonic acid may inhibit prostate cancer progression are unknown, because previous experimental studies have suggested that the arachidonic acid pathway may be implicated in prostate cancer development and progression through its involvement in inflammation and cell growth.^34^ Further research is therefore needed before conclusions on risk can be drawn.

We also found some evidence that both eicosenoic (22:1n‐9c) and eicosapentaenoic acid (20:5n‐3c) may be associated with a higher risk of death from prostate cancer. To the best of our knowledge, no previous prospective study has looked at the association between eicosenoic acid and prostate cancer risk, but this fatty acid is often found in similar foods as eicosapentaenoic acid (e.g. fish, and nuts and seeds).^35^ Circulating eicosapentaenoic acid concentrations have been associated with a higher risk of total prostate cancer in a pooled analysis of prospective studies^12^ and dietary intake of eicosapentaenoic acid was associated with a higher risk of advanced and fatal prostate cancer in the NIH‐AARP study.^36^ However, results from the Health Professionals Follow‐Up Study showed that a higher intake of eicosapentaenoic acid was associated with a lower risk of total and advanced prostate cancer.^32^

It was suggested in 1993 that α‐linolenic acid was positively associated with advanced prostate cancer.^37^ However, our results, the latest meta‐analysis from the WCRF/AIRC,^2^ and findings from a pooling study on circulating fatty acids^12^ do not support the hypothesis that higher intake of α‐linolenic acid increases the risk of prostate cancer. Previous prospective studies have also suggested that docosapentaenoic acid (22:5n‐3c) is involved in prostate cancer development,^12^ but we found no association between this fatty acid and prostate cancer risk. A Mendelian randomization analysis of data from up to 22,721 prostate cancer cases in the PRACTICAL consortia found no strong evidence for an association between several PUFAs, such as arachidonic acid, eicosapentaenoic acid, and docosapentaenoic acid, and overall and advanced prostate cancer risk.^38^

This study has some strengths and limitations that should be considered. The major strengths include the prospective design and the large sample size, which allowed investigation of fatty acids by stage and grade of prostate cancer tumors and death from prostate cancer. This study also had reliable identification of prostate cancer cases through cancer registries and/or verified medical records. The Gleason grade was based on data available from biopsies and surgical pathology, although there may be some misclassification because of changes in grading over time. The dietary questionnaires in all EPIC centers were validated and dietary intakes were calibrated using measures from a standardized 24‐h diet recall method to correct for over and under‐estimation of dietary intake.^15^

A limitation of the current study was the use of dietary fatty acids intake obtained from assessment questionnaires only at recruitment, which are subject to random measurement error and changes over time, and this would likely lead to an underestimation of true associations. Although we adjusted for multiple covariates, potential unmeasured and residual confounding cannot be excluded, including having a PSA test which was not available in our cohort. In addition, some of the associations observed might be due to chance because of the number of tests performed, and if we correct for multiple testing there would not be any significant results. Moreover, because some dietary fatty acids are highly correlated, it is difficult to disentangle their independent associations with prostate cancer. It is also unclear whether the risk differences observed in our study are attributable to the individual fatty acids or if they might be driven by other compound(s) in their food sources. Finally the fatty acids food sources may vary across the European countries included in this study, for example, the primary food that contributes to oleic acid intake in the south of Europe is olive oil, whereas in some populations in the north of Europe the primary food source is meat.^39^

In conclusion, intakes of individual fatty acids were not related to overall prostate cancer risk. However, our results suggest that higher intake of butyric acid may be associated with an increased risk of advanced prostate cancer. We also found a suggestive increased risk of fatal prostate cancer risk with higher intakes of eicosenoic acid and eicosapentaenoic acid. Further prospective studies and meta‐analyses are required to better understand the role of individual dietary fatty acids in prostate cancer development and progression.

## Supporting information

Appendix S1: Supplementary materials
