# Supplementary material for: Intake of individual fatty acids and risk of prostate cancer in the European prospective investigation into cancer and nutrition
Source: Int J Cancer. 2019 Mar 14;146(1):44–57. doi: 10.1002/ijc.32233 (PMC6899744; doi:10.1002/ijc.32233)
Supplement: Supplementary file 1 — Appendix S1: Supplementary materials [file IJC-146-44-s001.docx]

**Supplementary materials**

**Supplementary methods**

EPIC is a multicentre prospective study designed to investigate the relationships of dietary, lifestyle, genetic and biological factors with the incidence of cancer and other chronic diseases. It is conducted in 23 centres in 10 European countries: Denmark, France, Germany, Greece, Italy, The Netherlands, Norway, Spain, Sweden and the United Kingdom (UK). Participants were mostly recruited from the general population between 1992 and 2000. The full cohort comprises 519,978 participants including 153,457 men, most aged 35-70 years. The details of the study design used in the EPIC study have been described elsewhere (1).

In order to estimate the intakes of the individuals fatty acids, the EPIC Nutrient Database (ENDB) for the EPIC study was matched with the National Nutrient Database for Standard Reference of the United States (NNDSR; developed at the USDA) (2). The fatty acid intakes reported in this manuscript were obtained through this extra USDA matching and their quality has been confirmed through different quality controls. The first type of quality control includes the double-checking of the work performed by three dietitians among each other. The second type of quality controls includes the comparison between the nutrient values obtained through the ENDB procedures (matching with the national food composition databases) and this new USDA matching for the 28 food components that had already been matched with the EPIC food consumption data. Some correlation coefficients showing associations between some nutrients of interest for this specific study are shown in **Supplementary Tables 2**. Third, we compared between some nutrient values that overlap in both databases i.e. McCance and Widdowson food database and the USDA database), as some nutrients such as total energy intake (correlation coefficient 0.983), total fat (correlation coefficient 0.979), total SFAs (correlation coefficient 0.933), total MUFAs (correlation coefficient 0.967) or total PUFAs (correlation coefficient 0.897) are available in both databases.

Because isomers for MUFAs and PUFAs were not measured in every single food, we decided to use the undifferentiated values for palmitoleic acid (16:1n-7c), oleic acid (18:1n-9c), erucic acid (22:1n-9c), linoleic acid (18:2n-6c), α-linolenic acid (18:3n-3c), and arachidonic acid (20:4n-6c).

**References:**

1. Riboli E, Hunt KJ, Slimani N, Ferrari P, Norat T, Fahey M, Charrondiere UR, Hemon B, Casagrande C, Vignat J, et al. European prospective investigation into cancer and nutrition (EPIC): study populations and data collection. Public Health Nutrition 2002;5(6b):1113-24. doi: 10.1079/Phn2002394.

2. U.S. Department of Agriculture. Composition of Foods Raw, Processed, Prepared USDA National Nutrient Database for Standard Reference, Release 20. Maryland, US; 2008.

**Supplementary tables and figures**

| **Supplementary Table 1.** Intake of individual fatty acids of 142,239 men in EPIC (1992-2013) at baseline. | | | | | | | | | | | |
| --- | --- | --- | --- | --- | --- | --- | --- | --- | --- | --- | --- |
|  | **Fifths of observed SFAs intake** | | |  | **Fifths of observed MUFAs intake** | | |  | **Fifths of observed PUFAs intake** | | |
| **Observed intake (g/1000 Kcal)** | **1** | **3** | **5** |  | **1** | **3** | **5** |  | **1** | **3** | **5** |
| **Total fat** | 30.88 (6.07) | 38.60 (4.88) | 44.69 (5.21) |  | 29.55 (4.59) | 38.71 (3.59) | 45.71 (5.64) |  | 33.56 (6.87) | 38.11 (6.36) | 42.64 (6.21) |
| **Total SFAs** | 8.27 (1.35) | 12.45 (0.45) | 17.54 (2.22) |  | - | - | - |  | - | - | - |
| Butyric acid (4:0) | 0.136 (0.072) | 0.292 (0.086) | 0.567 (0.189) |  | - | - | - |  | - | - | - |
| Caproic acid (6:0) | 0.083 (0.044) | 0.180 (0.054) | 0.356 (0.122) |  | - | - | - |  | - | - | - |
| Caprylic acid (8:0) | 0.068 (0.035) | 0.137 (0.046) | 0.253 (0.088) |  | - | - | - |  | - | - | - |
| Capric acid (10:0) | 0.137 (0.068) | 0.278 (0.113) | 0.511 (0.206) |  | - | - | - |  | - | - | - |
| Lauric acid (12:0) | 0.209 (0.171) | 0.380 (0.235) | 0.627 (0.322) |  | - | - | - |  | - | - | - |
| Myristic acid (14:0) | 0.604 (0.213) | 1.131 (0.195) | 1.913 (0.438) |  | - | - | - |  | - | - | - |
| Pentadecanoic acid (15:0) | 0.016 (0.015) | 0.025 (0.023) | 0.029 (0.027) |  | - | - | - |  | - | - | - |
| Palmitic acid (16:0) | 4.76 (0.80) | 6.66 (0.46) | 8.80 (1.01) |  | - | - | - |  | - | - | - |
| Margaric acid (17:0) | 0.028 (0.012) | 0.042 (0.018) | 0.071 (0.037) |  | - | - | - |  | - | - | - |
| Stearic acid (18:0) | 1.98 (0.43) | 2.96 (0.41) | 4.01 (0.63) |  | - | - | - |  | - | - | - |
| Arachidic acid (20:0) | 0.055 (0.031) | 0.060 (0.032) | 0.056 (0.029) |  | - | - | - |  | - | - | - |
| Behenic acid (22:0) | 0.035 (0.042) | 0.038 (0.036) | 0.031 (0.040) |  | - | - | - |  | - | - | - |
| 4:0-10:0 | 0.425 (0.204) | 0.887 (0.264) | 1.686 (0.557) |  | - | - | - |  | - | - | - |
| 12:0-14:0 | 0.813 (0.332) | 1.513 (0.357) | 2.541 (0.659) |  | - | - | - |  | - | - | - |
|  |  |  |  |  |  |  |  |  |  |  |  |
| **Total MUFAs** | - | - | - |  | 10.21 (1.49) | 14.52 (0.46) | 21.53 (3.48) |  | - | - | - |
| Palmitoleic acid (16:1n-7c) | - | - | - |  | 0.409 (0.147) | 0.587 (0.158) | 0.716 (0.173) |  | - | - | - |
| Oleic acid (18:1n-9c) | - | - | - |  | 9.287 (1.418) | 13.172 (0.730) | 20.187 (3.613) |  | - | - | - |
| Eicosenoic acid (20:1n-9c) | - | - | - |  | 0.117 (0.084) | 0.154 (0.105) | 0.151 (0.095) |  | - | - | - |
| Erucic acid (22:1n-9c) | - | - | - |  | 0.070 (0.118) | 0.091 (0.146) | 0.052 (0.126) |  | - | - | - |
|  |  |  |  |  |  |  |  |  |  |  |  |
| **Total PUFAs** | - | - | - |  | - | - | - |  | 4.72 (0.56) | 6.92 (0.30) | 10.66 (1.84) |
| Linoleic acid (18:2n-6c) | - | - | - |  | - | - | - |  | 3.97 (0.53) | 5.94 (0.35) | 9.44 (1.86) |
| α-Linolenic acid (18:3n-3c) | - | - | - |  | - | - | - |  | 0.492 (0.090) | 0.686 (0.112) | 0.906 (0.254) |
| Arachidonic acid (20:4n-6c) | - | - | - |  | - | - | - |  | 0.057 (0.023) | 0.062 (0.029) | 0.058 (0.031) |
| Eicosapentaenoic acid (20:5n-3c) | - | - | - |  | - | - | - |  | 0.044 (0.047) | 0.052 (0.059) | 0.047 (0.059) |
| Docosapentaenoic acid (22:5n-3c) | - | - | - |  | - | - | - |  | 0.011 (0.009) | 0.012 (0.010) | 0.011 (0.010) |
| Docosahexaenoic acid (22:6n-3c) | - | - | - |  | - | - | - |  | 0.067 (0.064) | 0.075 (0.079) | 0.072 (0.083) |
| Values are means (SD) of grams per 1000 Kcal. Total fat, SFAs, MUFAs, PUFAs are not the sum of their parts due to missing data on some individual fatty acids | | | | | | | | | | | |
| Abbreviations: MUFAs, monounsaturated fatty acids; PUFAs, polyunsaturated fatty acids; SFAs saturated fatty acids. | | | | | | | | | | | |

| **Supplementary table 2A**. Pearson correlation coefficients between intakes of individual saturated fatty acids in 142,239 men in EPIC (1992-2013). | | | | | | | | | | | |
| --- | --- | --- | --- | --- | --- | --- | --- | --- | --- | --- | --- |
|  | **4:0** | **6:0** | **8:0** | **10:0** | **12:0** | **14:0** | **15:0** | **16:0** | **17:0** | **18:0** | **20:0** |
| **6:0** | 0.972 |  |  |  |  |  |  |  |  |  |  |
| **8:0** | 0.871 | 0.942 |  |  |  |  |  |  |  |  |  |
| **10:0** | 0.840 | 0.874 | 0.897 |  |  |  |  |  |  |  |  |
| **12:0** | 0.533 | 0.565 | 0.695 | 0.585 |  |  |  |  |  |  |  |
| **14:0** | 0.954 | 0.956 | 0.912 | 0.846 | 0.621 |  |  |  |  |  |  |
| **15:0** | 0.199 | 0.183 | 0.153 | 0.106 | 0.390 | 0.225 |  |  |  |  |  |
| **16:0** | 0.755 | 0.748 | 0.675 | 0.659 | 0.353 | 0.814 | 0.102 |  |  |  |  |
| **17:0** | 0.665 | 0.662 | 0.552 | 0.514 | 0.502 | 0.603 | 0.510 | 0.523 |  |  |  |
| **18:0** | 0.626 | 0.623 | 0.549 | 0.431 | 0.378 | 0.701 | 0.234 | 0.847 | 0.565 |  |  |
| **20:0** | -0.041 | -0.036 | 0.020 | 0.174 | 0.055 | -0.070 | -0.004 | 0.101 | 0.084 | -0.186 |  |
| **22:0** | -0.107 | -0.106 | -0.058 | -0.046 | 0.093 | -0.103 | 0.155 | -0.065 | 0.062 | -0.067 | 0.657 |
| All P values were < 0.001 | | | | | | | | | | | |

| **Supplementary table 2B**. Pearson correlation coefficients between intakes of individual monounsaturated fatty acids in 142,239 men in EPIC (1992-2013). | | | |
| --- | --- | --- | --- |
|  | **16:1n-7c** | **18:1n-9c** | **20:1n-9c** |
| **18:1n-9c** | 0.454 |  |  |
| **20:1n-9c** | 0.360 | 0.021 |  |
| **22:1n-9c** | 0.284 | -0.146 | 0.941 |
| All P values were < 0.001 | | | |

| **Supplementary table 2C**. Pearson correlation coefficients between intakes of individual polyunsaturated fatty acids in 142,239 men in EPIC (1992-2013). | | | | | | |
| --- | --- | --- | --- | --- | --- | --- |
|  | **18:2n-6c** | **18:3n-3c** | **20:4n-6c** | **20:5n-3c** | **22:5n-3c** | **22:6n-3c** |
| **18:3n-3c** | 0.609 |  |  |  |  |  |
| **20:4n-6c** | -0.022 | -0.158 |  |  |  |  |
| **20:5n-3c** | -0.062 | -0.058 | 0.481 |  |  |  |
| **22:5n-3c** | -0.081 | -0.085 | 0.523 | 0.872 |  |  |
| **22:6n-3c** | -0.049 | -0.090 | 0.530 | 0.968 | 0.883 |  |
| All P values were < 0.001 | | | | | | |

| **Supplementary Table 3.** Multivariable-adjusted hazard ratios (95 % CI) for prostate cancer per 1-SD increase of individual fatty acids intake in 142,239 men in EPIC (1992-2013). | | | | |
| --- | --- | --- | --- | --- |
|  |  | **Observed** | | |
|  | **No. cases** | **HR (95 % CI)**^1^ | **P trend**^2^ | **P het**^3^ |
| **Total Fat** |  |  |  |  |
| Total prostate cancer | 7036 | 0.99 (0.97 - 1.02) | 0.557 |  |
| Grade |  |  |  |  |
| Low | 3757 | 0.97 (0.93 - 1.00) | 0.064 |  |
| High | 726 | 1.05 (0.96 - 1.13) | 0.286 | 0.105 |
| Stage |  |  |  |  |
| Localized | 2641 | 0.98 (0.94 - 1.03) | 0.481 |  |
| Advanced | 1389 | 1.02 (0.96 - 1.08) | 0.584 | 0.502 |
| Fatal prostate cancer | 936 | 1.00 (0.93 - 1.07) | 0.893 |  |
| **Total SFAs** |  |  |  |  |
| Total prostate cancer | 7036 | 1.01 (0.98 - 1.03) | 0.679 |  |
| Grade |  |  |  |  |
| Low | 3757 | 0.98 (0.95 - 1.02) | 0.307 |  |
| High | 726 | 1.07 (0.99 - 1.16) | 0.075 | 0.091 |
| Stage |  |  |  |  |
| Localized | 2641 | 0.98 (0.94 - 1.02) | 0.259 |  |
| Advanced | 1389 | 1.05 (0.99 - 1.11) | 0.114 | 0.106 |
| Fatal prostate cancer | 936 | 1.02 (0.95 - 1.09) | 0.636 |  |
| **Butyric acid (4:0)** |  |  |  |  |
| Total prostate cancer | 7036 | 1.01 (0.99 - 1.04) | 0.354 |  |
| Grade |  |  |  |  |
| Low | 3757 | 0.99 (0.96 - 1.03) | 0.648 |  |
| High | 726 | 1.09 (1.01 - 1.17) | 0.035 | 0.156 |
| Stage |  |  |  |  |
| Localized | 2641 | 0.97 (0.93 - 1.01) | 0.130 |  |
| Advanced | 1389 | 1.07 (1.01 - 1.14) | 0.015 | 0.008 |
| Fatal prostate cancer | 936 | 1.03 (0.96 - 1.10) | 0.441 |  |
| **Caproic acid (6:0)** |  |  |  |  |
| Total prostate cancer | 7036 | 1.01 (0.98 - 1.03) | 0.632 |  |
| Grade |  |  |  |  |
| Low | 3757 | 0.98 (0.95 - 1.02) | 0.347 |  |
| High | 726 | 1.08 (1.00 - 1.16) | 0.057 | 0.126 |
| Stage |  |  |  |  |
| Localized | 2641 | 0.96 (0.92 - 1.00) | 0.073 |  |
| Advanced | 1389 | 1.06 (1.00 - 1.12) | 0.035 | 0.009 |
| Fatal prostate cancer | 936 | 1.03 (0.96 - 1.10) | 0.422 |  |
| **Caprylic acid (8:0)** |  |  |  |  |
| Total prostate cancer | 7036 | 1.00 (0.98 - 1.03) | 0.802 |  |
| Grade |  |  |  |  |
| Low | 3757 | 0.98 (0.94 - 1.01) | 0.177 |  |
| High | 726 | 1.05 (0.98 - 1.14) | 0.166 | 0.174 |
| Stage |  |  |  |  |
| Localized | 2641 | 0.96 (0.92 - 1.00) | 0.055 |  |
| Advanced | 1389 | 1.05 (0.99 - 1.11) | 0.093 | 0.014 |
| Fatal prostate cancer | 936 | 1.04 (0.97 - 1.11) | 0.286 |  |
| **Capric acid (10:0)** |  |  |  |  |
| Total prostate cancer | 7036 | 1.01 (0.98 - 1.04) | 0.647 |  |
| Grade |  |  |  |  |
| Low | 3757 | 0.98 (0.94 - 1.02) | 0.362 |  |
| High | 726 | 1.07 (0.98 - 1.17) | 0.118 | 0.208 |
| Stage |  |  |  |  |
| Localized | 2641 | 0.96 (0.92 - 1.01) | 0.122 |  |
| Advanced | 1389 | 1.06 (0.99 - 1.13) | 0.092 | 0.022 |
| Fatal prostate cancer | 936 | 1.03 (0.95 - 1.11) | 0.517 |  |
| **Lauric acid (12:0)** |  |  |  |  |
| Total prostate cancer | 7036 | 1.01 (0.98 - 1.04) | 0.447 |  |
| Grade |  |  |  |  |
| Low | 3757 | 0.99 (0.95 - 1.03) | 0.646 |  |
| High | 726 | 1.07 (0.99 - 1.15) | 0.109 | 0.108 |
| Stage |  |  |  |  |
| Localized | 2641 | 0.98 (0.92 - 1.03) | 0.383 |  |
| Advanced | 1389 | 1.05 (0.99 - 1.12) | 0.117 | 0.024 |
| Fatal prostate cancer | 936 | 1.05 (0.98 - 1.12) | 0.206 |  |
| **Myristic acid (14:0)** |  |  |  |  |
| Total prostate cancer | 7036 | 1.01 (0.98 - 1.03) | 0.534 |  |
| Grade |  |  |  |  |
| Low | 3757 | 0.98 (0.95 - 1.02) | 0.307 |  |
| High | 726 | 1.08 (1.00 - 1.17) | 0.051 | 0.126 |
| Stage |  |  |  |  |
| Localized | 2641 | 0.97 (0.93 - 1.01) | 0.125 |  |
| Advanced | 1389 | 1.06 (1.00 - 1.12) | 0.049 | 0.030 |
| Fatal prostate cancer | 936 | 1.03 (0.96 - 1.11) | 0.359 |  |
| **Pentadecanoic acid (15:0)** |  |  |  |  |
| Total prostate cancer | 7036 | 0.98 (0.95 - 1.00) | 0.097 |  |
| Grade |  |  |  |  |
| Low | 3757 | 0.98 (0.95 - 1.02) | 0.428 |  |
| High | 726 | 0.95 (0.87 - 1.03) | 0.224 | 0.268 |
| Stage |  |  |  |  |
| Localized | 2641 | 0.96 (0.91 - 1.01) | 0.099 |  |
| Advanced | 1389 | 0.99 (0.92 - 1.05) | 0.675 | 0.831 |
| Fatal prostate cancer | 936 | 0.99 (0.92 - 1.06) | 0.749 |  |
| **Palmitic acid (16:0)** |  |  |  |  |
| Total prostate cancer | 7036 | 1.00 (0.98 - 1.03) | 0.919 |  |
| Grade |  |  |  |  |
| Low | 3757 | 0.98 (0.95 - 1.02) | 0.256 |  |
| High | 726 | 1.06 (0.98 - 1.15) | 0.128 | 0.097 |
| Stage |  |  |  |  |
| Localized | 2641 | 0.98 (0.94 - 1.02) | 0.346 |  |
| Advanced | 1389 | 1.04 (0.98 - 1.10) | 0.237 | 0.222 |
| Fatal prostate cancer | 936 | 1.02 (0.95 - 1.09) | 0.609 |  |
| **Margaric acid (17:0)** |  |  |  |  |
| Total prostate cancer | 7036 | 0.99 (0.97 - 1.02) | 0.599 |  |
| Grade |  |  |  |  |
| Low | 3757 | 0.98 (0.95 - 1.02) | 0.368 |  |
| High | 726 | 1.05 (0.97 - 1.13) | 0.210 | 0.401 |
| Stage |  |  |  |  |
| Localized | 2641 | 0.97 (0.93 - 1.01) | 0.162 |  |
| Advanced | 1389 | 1.05 (0.99 - 1.11) | 0.114 | 0.027 |
| Fatal prostate cancer | 936 | 1.01 (0.94 - 1.08) | 0.745 |  |
| **Stearic acid (18:0)** |  |  |  |  |
| Total prostate cancer | 7036 | 1.00 (0.97 - 1.03) | 0.931 |  |
| Grade |  |  |  |  |
| Low | 3757 | 0.98 (0.94 - 1.02) | 0.286 |  |
| High | 726 | 1.06 (0.98 - 1.16) | 0.162 | 0.130 |
| Stage |  |  |  |  |
| Localized | 2641 | 0.98 (0.93 - 1.02) | 0.355 |  |
| Advanced | 1389 | 1.04 (0.98 - 1.11) | 0.206 | 0.212 |
| Fatal prostate cancer | 936 | 0.99 (0.92 - 1.07) | 0.886 |  |
| **Arachidic acid (20:0)** |  |  |  |  |
| Total prostate cancer | 7036 | 0.99 (0.95 - 1.03) | 0.535 |  |
| Grade |  |  |  |  |
| Low | 3757 | 0.99 (0.94 - 1.04) | 0.611 |  |
| High | 726 | 0.98 (0.88 - 1.10) | 0.790 | 0.639 |
| Stage |  |  |  |  |
| Localized | 2641 | 1.01 (0.95 - 1.07) | 0.860 |  |
| Advanced | 1389 | 0.95 (0.87 - 1.04) | 0.250 | 0.426 |
| Fatal prostate cancer | 936 | 0.95 (0.86 - 1.06) | 0.358 |  |
| **Behenic acid (22:0)** |  |  |  |  |
| Total prostate cancer | 7036 | 0.99 (0.96 - 1.02) | 0.595 |  |
| Grade |  |  |  |  |
| Low | 3757 | 0.99 (0.95 - 1.03) | 0.652 |  |
| High | 726 | 0.96 (0.86 - 1.08) | 0.516 | 0.277 |
| Stage |  |  |  |  |
| Localized | 2641 | 1.00 (0.94 - 1.07) | 0.979 |  |
| Advanced | 1389 | 0.94 (0.87 - 1.03) | 0.178 | 0.201 |
| Fatal prostate cancer | 936 | 0.95 (0.87 - 1.05) | 0.303 |  |
| **4:0-10:0** |  |  |  |  |
| Total prostate cancer | 7036 | 1.01 (0.98 - 1.03) | 0.547 |  |
| Grade |  |  |  |  |
| Low | 3757 | 0.98 (0.95 - 1.02) | 0.395 |  |
| High | 726 | 1.08 (0.99 - 1.16) | 0.067 | 0.160 |
| Stage |  |  |  |  |
| Localized | 2641 | 0.96 (0.92 - 1.01) | 0.093 |  |
| Advanced | 1389 | 1.06 (1.00 - 1.13) | 0.038 | 0.011 |
| Fatal prostate cancer | 936 | 1.03 (0.96 - 1.10) | 0.419 |  |
| **12:0-14:0** |  |  |  |  |
| Total prostate cancer | 7036 | 1.01 (0.98 - 1.04) | 0.477 |  |
| Grade |  |  |  |  |
| Low | 3757 | 0.98 (0.95 - 1.02) | 0.372 |  |
| High | 726 | 1.08 (1.00 - 1.17) | 0.048 | 0.110 |
| Stage |  |  |  |  |
| Localized | 2641 | 0.97 (0.93 - 1.01) | 0.160 |  |
| Advanced | 1389 | 1.06 (1.00 - 1.13) | 0.050 | 0.026 |
| Fatal prostate cancer | 936 | 1.04 (0.97 - 1.12) | 0.260 |  |
| Cox regression analysis. All models are stratified by centre and age at recruitment and adjusted for age (underlying time variable), educational level (no degree, degree or unknown), smoking status (never, former, current or unknown), marital status (married, not married, unknown), diabetes (yes, no, unknown), physical activity (inactive, moderately inactive, moderately active, active, unknown), height (<170, 170–174, 175–179, ≥ 180 cm or unknown), body mass index (<22.5, 22.5–24.9, 25–29.9, ≥ 30 kg/m^2^ or unknown), and total energy intake (fifths). | | | | |
| ^1^ HR (95% CI) estimated per 1-SD increase in fatty acids intake. | | | | |
| ^2^  Linear trends for HRs estimates over a continuous scale of the individual fatty acid. | | | | |
| ^3^ *P*-value from test for heterogeneity for the associations of intake the individual fatty acids with risk of prostate cancer categorized according to prostate tumour grade (low-intermediate or high) and stage (localized or advanced). | | | | |
| Low-intermediate grade (Gleason score of <8, or grade coded as well, moderately, or poorly differentiated). High grade (Gleason score of ≥ 8, or grade coded as undifferentiated). Localized stage (TNM staging score of T0-T2 and N0/Nx and M0, or stage coded in the recruitment centre as localized). Advanced stage (T3-T4 and/or N1-N3 and/or M1, and/or stage coded in the recruitment centre as metastatic). | | | | |
| Abbreviation: SFAs saturated fatty acids. | | | | |

| **Supplementary Table 4.** Multivariable-adjusted hazard ratios (95 % CI) for prostate cancer per 1-SD increase of individual fatty acids intake in 142,239 men in EPIC (1992-2013). | | | | |
| --- | --- | --- | --- | --- |
|  |  | **Observed** | | |
|  | **No. cases** | **HR (95 % CI)**^1^ | **P trend**^2^ | **P het**^3^ |
| **Total MUFAs** |  |  |  |  |
| Total prostate cancer | 7036 | 0.99 (0.95 - 1.02) | 0.470 |  |
| Grade |  |  |  |  |
| Low | 3757 | 0.97 (0.93 - 1.02) | 0.242 |  |
| High | 726 | 1.02 (0.92 - 1.14) | 0.691 | 0.270 |
| Stage |  |  |  |  |
| Localized | 2641 | 1.00 (0.95 - 1.06) | 0.896 |  |
| Advanced | 1389 | 1.00 (0.92 - 1.08) | 1.000 | 0.941 |
| Fatal prostate cancer | 936 | 0.97 (0.88 - 1.07) | 0.564 |  |
| **Palmitoleic acid (16:1n-7c)** |  |  |  |  |
| Total prostate cancer | 7036 | 1.01 (0.98 - 1.04) | 0.719 |  |
| Grade |  |  |  |  |
| Low | 3757 | 0.99 (0.95 - 1.03) | 0.693 |  |
| High | 726 | 1.05 (0.95 - 1.15) | 0.358 | 0.232 |
| Stage |  |  |  |  |
| Localized | 2641 | 1.01 (0.96 - 1.06) | 0.768 |  |
| Advanced | 1389 | 1.02 (0.96 - 1.10) | 0.521 | 0.929 |
| Fatal prostate cancer | 936 | 1.04 (0.95 - 1.13) | 0.392 |  |
| **Oleic acid (18:1n-9c)** |  |  |  |  |
| Total prostate cancer | 7036 | 0.99 (0.96 - 1.02) | 0.540 |  |
| Grade |  |  |  |  |
| Low | 3757 | 0.97 (0.93 - 1.02) | 0.289 |  |
| High | 726 | 1.01 (0.91 - 1.13) | 0.799 | 0.356 |
| Stage |  |  |  |  |
| Localized | 2641 | 1.00 (0.94 - 1.06) | 0.989 |  |
| Advanced | 1389 | 1.00 (0.92 - 1.09) | 0.941 | 0.732 |
| Fatal prostate cancer | 936 | 0.97 (0.88 - 1.07) | 0.610 |  |
| **Eicosenoic acid (20:1n-9c)** |  |  |  |  |
| Total prostate cancer | 7036 | 1.00 (0.98 - 1.02) | 0.954 |  |
| Grade |  |  |  |  |
| Low | 3757 | 0.98 (0.95 - 1.02) | 0.367 |  |
| High | 726 | 1.04 (0.97 - 1.10) | 0.290 | 0.047 |
| Stage |  |  |  |  |
| Localized | 2641 | 1.00 (0.96 - 1.03) | 0.797 |  |
| Advanced | 1389 | 1.01 (0.97 - 1.06) | 0.573 | 0.595 |
| Fatal prostate cancer | 936 | 1.04 (0.98 - 1.10) | 0.165 |  |
| **Erucic acid (22:1n-9c)** |  |  |  |  |
| Total prostate cancer | 7036 | 1.00 (0.98 - 1.03) | 0.663 |  |
| Grade |  |  |  |  |
| Low | 3757 | 0.99 (0.95 - 1.02) | 0.431 |  |
| High | 726 | 1.04 (0.98 - 1.11) | 0.194 | 0.042 |
| Stage |  |  |  |  |
| Localized | 2641 | 0.99 (0.95 - 1.03) | 0.680 |  |
| Advanced | 1389 | 1.02 (0.98 - 1.07) | 0.328 | 0.443 |
| Fatal prostate cancer | 936 | 1.04 (0.99 - 1.10) | 0.109 |  |
| Cox regression analysis. All models are stratified by centre and age at recruitment and adjusted for age (underlying time variable), educational level (no degree, degree or unknown), smoking status (never, former, current or unknown), marital status (married, not married, unknown), diabetes (yes, no, unknown), physical activity (inactive, moderately inactive, moderately active, active, unknown), height (<170, 170–174, 175–179, ≥ 180 cm or unknown), body mass index (<22.5, 22.5–24.9, 25–29.9, ≥ 30 kg/m^2^ or unknown), and total energy intake (fifths). | | | | |
| ^1^ HR (95% CI) estimated per 1-SD increase in fatty acids intake. | | | | |
| ^2^  Linear trends for HRs estimates over a continuous scale of the individual fatty acid. | | | | |
| ^3^ *P*-value from test for heterogeneity for the associations of intake the individual fatty acids with risk of prostate cancer categorized according to prostate tumour grade (low-intermediate or high) and stage (localized or advanced). | | | | |
| Low-intermediate grade (Gleason score of <8, or grade coded as well, moderately, or poorly differentiated). High grade (Gleason score of ≥ 8, or grade coded as undifferentiated). Localized stage (TNM staging score of T0-T2 and N0/Nx and M0, or stage coded in the recruitment centre as localized). Advanced stage (T3-T4 and/or N1-N3 and/or M1, and/or stage coded in the recruitment centre as metastatic). | | | | |
| Abbreviations: MUFAs, monounsaturated fatty acids. | | | | |

| **Supplementary Table 5.** Multivariable-adjusted hazard ratios (95 % CI) for prostate cancer per 1-SD increase of individual fatty acids intake in 142,239 men in EPIC (1992-2013). | | | | |
| --- | --- | --- | --- | --- |
|  |  | **Observed** | | |
|  | **No. cases** | **HR (95 % CI)**^1^ | **P trend**^2^ | **P het**^3^ |
| **Total PUFAs** |  |  |  |  |
| Total prostate cancer | 7036 | 0.99 (0.96 - 1.01) | 0.362 |  |
| Grade |  |  |  |  |
| Low | 3757 | 0.97 (0.93 - 1.00) | 0.080 |  |
| High | 726 | 1.00 (0.92 - 1.08) | 0.956 | 0.413 |
| Stage |  |  |  |  |
| Localized | 2641 | 0.99 (0.95 - 1.03) | 0.670 |  |
| Advanced | 1389 | 0.98 (0.92 - 1.04) | 0.529 | 0.621 |
| Fatal prostate cancer | 936 | 0.99 (0.92 - 1.07) | 0.825 |  |
| **Linoleic acid (18:2n-6c)** |  |  |  |  |
| Total prostate cancer | 7036 | 0.99 (0.96 - 1.01) | 0.339 |  |
| Grade |  |  |  |  |
| Low | 3757 | 0.96 (0.93 - 1.00) | 0.058 |  |
| High | 726 | 1.00 (0.92 - 1.09) | 0.991 | 0.342 |
| Stage |  |  |  |  |
| Localized | 2641 | 0.99 (0.95 - 1.03) | 0.560 |  |
| Advanced | 1389 | 0.98 (0.92 - 1.04) | 0.515 | 0.709 |
| Fatal prostate cancer | 936 | 0.99 (0.92 - 1.07) | 0.849 |  |
| **α-Linolenic acid (18:3n-3c)** |  |  |  |  |
| Total prostate cancer | 7036 | 0.99 (0.96 - 1.01) | 0.400 |  |
| Grade |  |  |  |  |
| Low | 3757 | 0.98 (0.94 - 1.01) | 0.202 |  |
| High | 726 | 1.00 (0.93 - 1.09) | 0.908 | 0.487 |
| Stage |  |  |  |  |
| Localized | 2641 | 0.98 (0.94 - 1.03) | 0.390 |  |
| Advanced | 1389 | 1.01 (0.95 - 1.07) | 0.749 | 0.672 |
| Fatal prostate cancer | 936 | 1.00 (0.94 - 1.07) | 0.978 |  |
| **Arachidonic acid (20:4n-6c)** |  |  |  |  |
| Total prostate cancer | 7036 | 1.00 (0.97 - 1.03) | 0.826 |  |
| Grade |  |  |  |  |
| Low | 3757 | 0.99 (0.95 - 1.03) | 0.688 |  |
| High | 726 | 0.95 (0.87 - 1.04) | 0.275 | 0.655 |
| Stage |  |  |  |  |
| Localized | 2641 | 1.03 (0.99 - 1.08) | 0.167 |  |
| Advanced | 1389 | 0.94 (0.88 - 1.00) | 0.066 | 0.029 |
| Fatal prostate cancer | 936 | 1.02 (0.95 - 1.10) | 0.592 |  |
| **Eicosapentaenoic acid (20:5n-3c)** |  |  |  |  |
| Total prostate cancer | 7036 | 1.01 (0.99 - 1.04) | 0.285 |  |
| Grade |  |  |  |  |
| Low | 3757 | 1.01 (0.98 - 1.05) | 0.519 |  |
| High | 726 | 0.99 (0.92 - 1.07) | 0.848 | 0.798 |
| Stage |  |  |  |  |
| Localized | 2641 | 1.01 (0.97 - 1.06) | 0.490 |  |
| Advanced | 1389 | 1.00 (0.94 - 1.05) | 0.890 | 0.659 |
| Fatal prostate cancer | 936 | 1.04 (0.98 - 1.10) | 0.231 |  |
| **Docosapentaenoic acid (22:5n-3c)** | |  |  |  |
| Total prostate cancer | 7036 | 1.01 (0.99 - 1.03) | 0.484 |  |
| Grade |  |  |  |  |
| Low | 3757 | 1.01 (0.98 - 1.04) | 0.586 |  |
| High | 726 | 0.97 (0.89 - 1.05) | 0.440 | 0.577 |
| Stage |  |  |  |  |
| Localized | 2641 | 1.02 (0.98 - 1.06) | 0.314 |  |
| Advanced | 1389 | 0.98 (0.92 - 1.04) | 0.439 | 0.409 |
| Fatal prostate cancer | 936 | 1.00 (0.94 - 1.07) | 0.959 |  |
| **Docosahexaenoic acid (22:6n-3c)** |  |  |  |  |
| Total prostate cancer | 7036 | 1.01 (0.99 - 1.03) | 0.425 |  |
| Grade |  |  |  |  |
| Low | 3757 | 1.01 (0.97 - 1.05) | 0.556 |  |
| High | 726 | 0.97 (0.89 - 1.06) | 0.495 | 0.520 |
| Stage |  |  |  |  |
| Localized | 2641 | 1.01 (0.97 - 1.06) | 0.529 |  |
| Advanced | 1389 | 0.99 (0.93 - 1.05) | 0.677 | 0.767 |
| Fatal prostate cancer | 936 | 1.03 (0.97 - 1.09) | 0.356 |  |
| Cox regression analysis. All models are stratified by centre and age at recruitment and adjusted for age (underlying time variable), educational level (no degree, degree or unknown), smoking status (never, former, current or unknown), marital status (married, not married, unknown), diabetes (yes, no, unknown), physical activity (inactive, moderately inactive, moderately active, active, unknown), height (<170, 170–174, 175–179, ≥ 180 cm or unknown), body mass index (<22.5, 22.5–24.9, 25–29.9, ≥ 30 kg/m^2^ or unknown), and total energy intake (fifths). | | | | |
| ^1^ HR (95% CI) estimated per 1-SD increase in fatty acids intake. | | | | |
| ^2^  Linear trends for HRs estimates over a continuous scale of the individual fatty acid. | | | | |
| ^3^ *P*-value from test for heterogeneity for the associations of intake the individual fatty acids with risk of prostate cancer categorized according to prostate tumour grade (low-intermediate or high) and stage (localized or advanced). | | | | |
| Low-intermediate grade (Gleason score of <8, or grade coded as well, moderately, or poorly differentiated). High grade (Gleason score of ≥ 8, or grade coded as undifferentiated). Localized stage (TNM staging score of T0-T2 and N0/Nx and M0, or stage coded in the recruitment centre as localized). Advanced stage (T3-T4 and/or N1-N3 and/or M1, and/or stage coded in the recruitment centre as metastatic). | | | | |
| Abbreviation: PUFAs, polyunsaturated fatty acids. | | | | |

| **Supplementary Table 6.** Multivariable-adjusted hazard ratios (95 % CI) for total prostate cancer by fifths of observed individual fatty acids intake in 142,239 men in EPIC (1992-2013). | | | | | |
| --- | --- | --- | --- | --- | --- |
|  | **Fifths of observed intake** | | | | |
|  | **1** | **2** | **3** | **4** | **5** |
| **Total fat, g/1000 Kcal** | ≤ 32.6 | >32.6 to ≤ 36.5 | >36.5 to ≤ 39.9 | >39.9 to ≤ 43.9 | >43.9 |
| Cases, *n* | 1557 | 1541 | 1423 | 1341 | 1174 |
| HR (95% CI) | 1 ref | 1.02 (0.95 - 1.09) | 0.98 (0.91 - 1.06) | 0.97 (0.90 - 1.05) | 0.99 (0.91 - 1.07) |
| Adjusted HR (95% CI) ^1^ | 1 ref | 1.01 (0.94 - 1.09) | 0.98 (0.91 - 1.05) | 0.97 (0.90 - 1.04) | 0.99 (0.92 - 1.08) |
| **Total SFAs, g/1000 Kcal** | ≤ 12.0 | >12.0 to ≤ 13.7 | >13.7 to ≤ 15.3 | >15.3 to ≤ 17.7 | >17.7 |
| Cases, *n* | 1373 | 1478 | 1487 | 1380 | 1318 |
| HR (95% CI) | 1 ref | 1.06 (0.98 - 1.14) | 1.09 (1.01 - 1.18) | 1.05 (0.97 - 1.14) | 1.03 (0.95 - 1.12) |
| Adjusted HR (95% CI) ^1^ | 1 ref | 1.05 (0.97 - 1.13) | 1.09 (1.01 - 1.17) | 1.05 (0.97 - 1.14) | 1.03 (0.94 - 1.11) |
| **Butyric acid (4:0), g/1000 Kcal** | ≤ 0.167 | >0.167 to ≤ 0.251 | >0.251 to ≤ 0.332 | >0.332 to ≤ 0.448 | > 0.448 |
| Cases, *n* | 1436 | 1508 | 1488 | 1351 | 1253 |
| HR (95% CI) | 1 ref | 1.05 (0.97 - 1.13) | 1.10 (1.02 - 1.19) | 1.11 (1.02 - 1.20) | 1.05 (0.96 - 1.14) |
| Adjusted HR (95% CI) ^1^ | 1 ref | 1.04 (0.96 - 1.12) | 1.09 (1.01 - 1.18) | 1.09 (1.01 - 1.18) | 1.03 (0.95 - 1.12) |
| **Caproic acid (6:0), g/1000 Kcal** | ≤ 0.103 | >0.103 to ≤ 0.153 | >0.153 to ≤ 0.205 | >0.205 to ≤ 0.278 | > 0.278 |
| Cases, *n* | 1470 | 1509 | 1481 | 1336 | 1240 |
| HR (95% CI) | 1 ref | 1.05 (0.97 - 1.12) | 1.11 (1.03 - 1.19) | 1.11 (1.03 - 1.20) | 1.05 (0.97 - 1.14) |
| Adjusted HR (95% CI) ^1^ | 1 ref | 1.04 (0.96 - 1.12) | 1.09 (1.02 - 1.18) | 1.10 (1.02 - 1.19) | 1.03 (0.95 - 1.12) |
| **Caprylic acid (8:0), g/1000 Kcal** | ≤ 0.079 | >0.167 to ≤ 0.251 | >0.251 to ≤ 0.332 | >0.332 to ≤ 0.448 | > 0.448 |
| Cases, *n* | 1506 | 1529 | 1478 | 1317 | 1206 |
| HR (95% CI) | 1 ref | 1.05 (0.98 - 1.13) | 1.11 (1.04 - 1.20) | 1.11 (1.03 - 1.20) | 1.04 (0.96 - 1.13) |
| Adjusted HR (95% CI) ^1^ | 1 ref | 1.04 (0.97 - 1.12) | 1.10 (1.02 - 1.18) | 1.09 (1.01 - 1.18) | 1.02 (0.94 - 1.11) |
| **Capric acid (10:0), g/1000 Kcal** | ≤ 0.158 | >0.158 to ≤ 0.226 | >0.226 to ≤ 0.298 | >0.298 to ≤ 0.410 | > 0.410 |
| Cases, *n* | 1541 | 1586 | 1542 | 1322 | 1045 |
| HR (95% CI) | 1 ref | 1.05 (0.98 - 1.13) | 1.13 (1.05 - 1.22) | 1.11 (1.03 - 1.20) | 1.06 (0.97 - 1.15) |
| Adjusted HR (95% CI) ^1^ | 1 ref | 1.05 (0.97 - 1.12) | 1.12 (1.04 - 1.20) | 1.09 (1.01 - 1.18) | 1.04 (0.95 - 1.13) |
| **Lauric acid (12:0), g/1000 Kcal** | ≤ 0.198 | >0.198 to ≤ 0.289 | >0.289 to ≤ 0.385 | >0.385 to ≤ 0.535 | > 0.535 |
| Cases, *n* | 1562 | 1623 | 1422 | 1301 | 1128 |
| HR (95% CI) | 1 ref | 1.08 (1.01 - 1.16) | 1.08 (1.00 - 1.16) | 1.16 (1.07 - 1.25) | 1.01 (0.92 - 1.11) |
| Adjusted HR (95% CI) ^1^ | 1 ref | 1.07 (0.99 - 1.15) | 1.06 (0.98 - 1.14) | 1.13 (1.04 - 1.23) | 0.98 (0.89 - 1.08) |
| **Myristic acid (14:0), g/1000 Kcal** | ≤ 0.763 | >0.763 to ≤ 1.012 | >1.012 to ≤ 1.250 | >1.250 to ≤ 1.574 | > 1.574 |
| Cases, *n* | 1332 | 1513 | 1489 | 1432 | 1270 |
| HR (95% CI) | 1 ref | 1.08 (1.00 - 1.16) | 1.10 (1.02 - 1.19) | 1.13 (1.04 - 1.22) | 1.05 (0.97 - 1.14) |
| Adjusted HR (95% CI) ^1^ | 1 ref | 1.07 (0.99 - 1.16) | 1.09 (1.01 - 1.18) | 1.11 (1.03 - 1.20) | 1.03 (0.95 - 1.12) |
| **Pentadecanoic acid (15:0), g/1000 Kcal** | ≤ 0.007 | >0.007 to ≤ 0.012 | >0.012 to ≤ 0.021 | >0.021 to ≤ 0.038 | > 0.038 |
| Cases, *n* | 1172 | 1625 | 1499 | 1407 | 1333 |
| HR (95% CI) | 1 ref | 1.07 (0.99 - 1.16) | 1.03 (0.95 - 1.12) | 1.05 (0.96 - 1.14) | 0.97 (0.89 - 1.06) |
| Adjusted HR (95% CI) ^1^ | 1 ref | 1.07 (0.99 - 1.16) | 1.03 (0.95 - 1.12) | 1.05 (0.96 - 1.14) | 0.98 (0.89 - 1.07) |
| **Palmitic acid (16:0), g/1000 Kcal** | ≤ 5.487 | >5.487 to ≤ 6.308 | >6.308 to ≤ 7.027 | >7.027 to ≤ 7.900 | > 7.900 |
| Cases, *n* | 1468 | 1471 | 1490 | 1276 | 1331 |
| HR (95% CI) | 1 ref | 0.99 (0.92 - 1.07) | 1.07 (0.99 - 1.15) | 0.98 (0.91 - 1.06) | 1.01 (0.93 - 1.09) |
| Adjusted HR (95% CI) ^1^ | 1 ref | 0.99 (0.92 - 1.06) | 1.07 (0.99 - 1.15) | 0.98 (0.90 - 1.06) | 1.01 (0.93 - 1.10) |
| **Margaric acid (17:0), g/1000 Kcal** | ≤ 0.024 | >0.024 to ≤ 0.033 | >0.033 to ≤ 0.044 | >0.044 to ≤ 0.062 | > 0.062 |
| Cases, *n* | 1592 | 1500 | 1387 | 1302 | 1255 |
| HR (95% CI) | 1 ref | 1.06 (0.99 - 1.14) | 1.06 (0.99 - 1.14) | 1.08 (1.00 - 1.17) | 1.01 (0.93 - 1.10) |
| Adjusted HR (95% CI) ^1^ | 1 ref | 1.07 (0.99 - 1.15) | 1.07 (0.99 - 1.15) | 1.09 (1.01 - 1.18) | 1.01 (0.93 - 1.10) |
| **Stearic acid (18:0), g/1000 Kcal** | ≤ 2.258 | >2.258 to ≤ 2.728 | >2.728 to ≤ 3.160 | >3.160 to ≤ 3.658 | > 3.658 |
| Cases, *n* | 1124 | 1363 | 1526 | 1556 | 1467 |
| HR (95% CI) | 1 ref | 1.07 (0.98 - 1.16) | 1.05 (0.96 - 1.14) | 1.05 (0.96 - 1.14) | 1.00 (0.92 - 1.10) |
| Adjusted HR (95% CI) ^1^ | 1 ref | 1.06 (0.98 - 1.15) | 1.04 (0.96 - 1.13) | 1.05 (0.96 - 1.14) | 1.01 (0.92 - 1.11) |
| **Arachidic acid (20:0), g/1000 Kcal** | ≤ 0.033 | >0.033 to ≤ 0.045 | >0.045 to ≤ 0.058 | >0.058 to ≤ 0.078 | > 0.078 |
| Cases, *n* | 1878 | 1616 | 1513 | 1323 | 706 |
| HR (95% CI) | 1 ref | 1.00 (0.93 - 1.07) | 0.98 (0.91 - 1.05) | 1.00 (0.93 - 1.08) | 0.94 (0.85 - 1.04) |
| Adjusted HR (95% CI) ^1^ | 1 ref | 1.00 (0.93 - 1.07) | 0.98 (0.91 - 1.05) | 0.99 (0.92 - 1.08) | 0.93 (0.84 - 1.04) |
| **Behenic acid (22:0), g/1000 Kcal** | ≤ 0.014 | >0.014 to ≤ 0.022 | >0.022 to ≤ 0.032 | >0.032 to ≤ 0.047 | > 0.047 |
| Cases, *n* | 1669 | 1448 | 1523 | 1422 | 974 |
| HR (95% CI) | 1 ref | 1.01 (0.94 - 1.09) | 1.05 (0.97 - 1.13) | 1.02 (0.94 - 1.11) | 0.99 (0.91 - 1.09) |
| Adjusted HR (95% CI) ^1^ | 1 ref | 1.01 (0.94 - 1.09) | 1.05 (0.97 - 1.13) | 1.02 (0.94 - 1.11) | 0.99 (0.91 - 1.09) |
| **4:0-10:0, g/1000 Kcal** | ≤ 0.517 | >0.517 to ≤ 0.753 | >0.753 to ≤ 0.995 | >0.995 to ≤ 1.346 | > 1.346 |
| Cases, *n* | 1500 | 1545 | 1520 | 1307 | 1164 |
| HR (95% CI) | 1 ref | 1.05 (0.97 - 1.12) | 1.11 (1.03 - 1.19) | 1.08 (1.00 - 1.17) | 1.05 (0.97 - 1.15) |
| Adjusted HR (95% CI) ^1^ | 1 ref | 1.04 (0.97 - 1.12) | 1.09 (1.02 - 1.18) | 1.07 (0.99 - 1.15) | 1.04 (0.95 - 1.12) |
| **12:0-14:0, g/1000 Kcal** | ≤ 0.988 | >0.988 to ≤ 1.328 | >1.328 to ≤ 1.663 | >1.663 to ≤ 2.120 | > 2.120 |
| Cases, *n* | 1384 | 1551 | 1518 | 1372 | 1211 |
| HR (95% CI) | 1 ref | 1.08 (1.00 - 1.17) | 1.13 (1.04 - 1.22) | 1.12 (1.03 - 1.21) | 1.05 (0.96 - 1.14) |
| Adjusted HR (95% CI) ^1^ | 1 ref | 1.08 (1.00 - 1.16) | 1.11 (1.03 - 1.20) | 1.10 (1.02 - 1.19) | 1.03 (0.95 - 1.12) |
|  |  |  |  |  |  |
| **Total MUFAs, g/1000 Kcal** | ≤ 12.0 | >12.0 to ≤ 13.7 | >13.7 to ≤ 15.3 | >15.3 to ≤ 17.7 | >43.9 |
| Cases, *n* | 1573 | 1602 | 1481 | 1396 | 984 |
| HR (95% CI) | 1 ref | 1.02 (0.95 - 1.09) | 0.96 (0.89 - 1.03) | 0.96 (0.89 - 1.03) | 0.95 (0.87 - 1.05) |
| Adjusted HR (95% CI) ^1^ | 1 ref | 1.01 (0.94 - 1.08) | 0.96 (0.89 - 1.03) | 0.96 (0.89 - 1.04) | 0.97 (0.88 - 1.06) |
| **Palmitoleic acid (16:1n-7c), g/1000 Kcal** | ≤ 0.410 | >0.410 to ≤ 0.532 | >0.532 to ≤ 0.626 | >0.626 to ≤ 0.732 | >43.9 |
| Cases, *n* | 1373 | 1443 | 1399 | 1408 | 1413 |
| HR (95% CI) | 1 ref | 0.99 (0.91 - 1.08) | 0.99 (0.90 - 1.09) | 1.03 (0.94 - 1.13) | 0.99 (0.89 - 1.08) |
| Adjusted HR (95% CI) ^1^ | 1 ref | 1.00 (0.91 - 1.08) | 1.00 (0.91 - 1.09) | 1.04 (0.95 - 1.15) | 1.01 (0.92 - 1.12) |
| **Oleic acid (18:1n-9c), g/1000 Kcal** | ≤ 10.796 | >10.796 to ≤ 12.428 | >12.428 to ≤ 13.968 | >13.969 to ≤ 16.361 | > 16.361 |
| Cases, *n* | 1649 | 1591 | 1489 | 1334 | 973 |
| HR (95% CI) | 1 ref | 0.98 (0.92 - 1.05) | 0.97 (0.90 - 1.04) | 0.92 (0.85 - 0.99) | 0.97 (0.89 - 1.07) |
| Adjusted HR (95% CI) ^1^ | 1 ref | 0.98 (0.91 - 1.05) | 0.96 (0.90 - 1.03) | 0.92 (0.86 - 1.00) | 0.98 (0.90 - 1.08) |
| **Eicosenoic acid (20:1n-9c), g/1000 Kcal** | ≤ 0.088 | >0.088 to ≤ 0.109 | >0.109 to ≤ 0.132 | >0.132 to ≤ 0.175 | > 0.175 |
| Cases, *n* | 1283 | 1246 | 1201 | 1412 | 1894 |
| HR (95% CI) | 1 ref | 1.02 (0.94 - 1.10) | 1.00 (0.92 - 1.08) | 1.01 (0.94 - 1.10) | 1.03 (0.95 - 1.11) |
| Adjusted HR (95% CI) ^1^ | 1 ref | 1.02 (0.94 - 1.10) | 1.00 (0.93 - 1.09) | 1.02 (0.94 - 1.11) | 1.03 (0.96 - 1.12) |
| **Erucic acid (22:1n-9c), g/1000 Kcal** | ≤ 0.007 | >0.007 to ≤ 0.018 | >0.018 to ≤ 0.046 | >0.046 to ≤ 0.113 | > 0.113 |
| Cases, *n* | 914 | 1107 | 1350 | 1763 | 1902 |
| HR (95% CI) | 1 ref | 0.99 (0.90 - 1.08) | 0.95 (0.87 - 1.04) | 1.07 (0.98 - 1.17) | 1.02 (0.93 - 1.11) |
| Adjusted HR (95% CI) ^1^ | 1 ref | 0.98 (0.90 - 1.08) | 0.95 (0.87 - 1.04) | 1.07 (0.97 - 1.17) | 1.01 (0.92 - 1.11) |
|  |  |  |  |  |  |
| **Total PUFAs, g/1000 Kcal** | ≤ 5.4 | >5.4 to ≤ 6.4 | >6.4 to ≤ 7.5 | >7.5 to ≤ 8.8 | >8.8 |
| Cases, *n* | 1291 | 1328 | 1449 | 1585 | 1383 |
| HR (95% CI) | 1 ref | 1.04 (0.96 - 1.13) | 1.00 (0.92 - 1.08) | 1.02 (0.94 - 1.10) | 0.98 (0.90 - 1.07) |
| Adjusted HR (95% CI) ^1^ | 1 ref | 1.04 (0.96 - 1.12) | 1.00 (0.92 - 1.08) | 1.02 (0.94 - 1.10) | 0.98 (0.90 - 1.07) |
| **Linoleic acid (18:2n-6c), g/1000 Kcal** | ≤ 4.596 | >4.596 to ≤ 5.478 | >5.478 to ≤ 6.421 | >6.422 to ≤ 7.712 | > 7.712 |
| Cases, *n* | 1354 | 1329 | 1476 | 1578 | 1299 |
| HR (95% CI) | 1 ref | 1.01 (0.93 - 1.09) | 1.01 (0.93 - 1.09) | 1.03 (0.95 - 1.11) | 0.98 (0.90 - 1.06) |
| Adjusted HR (95% CI) ^1^ | 1 ref | 1.01 (0.93 - 1.09) | 1.01 (0.93 - 1.09) | 1.03 (0.95 - 1.11) | 0.98 (0.90 - 1.06) |
| **α-Linolenic acid (18:3n-3c), g/1000 Kcal** | ≤ 0.519 | >0.519 to ≤ 0.617 | >0.617 to ≤ 0.718 | >0.718 to ≤ 0.850 | > 0.850 |
| Cases, *n* | 1359 | 1299 | 1367 | 1461 | 1550 |
| HR (95% CI) | 1 ref | 1.04 (0.96 - 1.12) | 1.04 (0.96 - 1.13) | 1.02 (0.94 - 1.11) | 0.97 (0.89 - 1.05) |
| Adjusted HR (95% CI) ^1^ | 1 ref | 1.03 (0.95 - 1.11) | 1.04 (0.96 - 1.12) | 1.02 (0.94 - 1.10) | 0.97 (0.89 - 1.05) |
| **Arachidonic acid (20:4n-6c), g/1000 Kcal** | ≤ 0.037 | >0.037 to ≤ 0.050 | >0.050 to ≤ 0.063 | >0.063 to ≤ 0.081 | > 0.081 |
| Cases, *n* | 1239 | 1247 | 1456 | 1537 | 1557 |
| HR (95% CI) | 1 ref | 0.95 (0.87 - 1.03) | 0.99 (0.91 - 1.08) | 0.94 (0.86 - 1.03) | 0.92 (0.84 - 1.01) |
| Adjusted HR (95% CI) ^1^ | 1 ref | 0.95 (0.88 - 1.04) | 1.01 (0.93 - 1.10) | 0.97 (0.88 - 1.06) | 0.96 (0.87 - 1.05) |
| **Eicosapentaenoic acid (20:5n-3c), g/1000 Kcal** | ≤ 0.010 | >0.010 to ≤ 0.022 | >0.022 to ≤ 0.039 | >0.039 to ≤ 0.076 | > 0.076 |
| Cases, *n* | 1000 | 1097 | 1334 | 1581 | 2024 |
| HR (95% CI) | 1 ref | 0.99 (0.91 - 1.09) | 1.05 (0.96 - 1.14) | 1.08 (0.99 - 1.17) | 1.08 (0.99 - 1.18) |
| Adjusted HR (95% CI) ^1^ | 1 ref | 0.99 (0.91 - 1.08) | 1.05 (0.96 - 1.14) | 1.08 (0.99 - 1.18) | 1.08 (0.99 - 1.18) |
| **Docosapentaenoic acid (22:5n-3c), g/1000 Kcal** | ≤ 0.004 | >0.004 to ≤ 0.007 | >0.007 to ≤ 0.011 | >0.011 to ≤ 0.017 | > 0.017 |
| Cases, *n* | 820 | 1240 | 1471 | 1664 | 1841 |
| HR (95% CI) | 1 ref | 1.05 (0.95 - 1.15) | 1.07 (0.97 - 1.17) | 1.11 (1.01 - 1.21) | 1.06 (0.97 - 1.16) |
| Adjusted HR (95% CI) ^1^ | 1 ref | 1.05 (0.96 - 1.15) | 1.07 (0.98 - 1.18) | 1.12 (1.02 - 1.22) | 1.07 (0.97 - 1.17) |
| **Docosahexaenoic acid (22:6n-3c), g/1000 Kcal** | ≤ 0.018 | >0.018 to ≤ 0.037 | >0.037 to ≤ 0.061 | >0.061 to ≤ 0.114 | > 0.114 |
| Cases, *n* | 973 | 1146 | 1380 | 1615 | 1922 |
| HR (95% CI) | 1 ref | 1.04 (0.95 - 1.14) | 1.09 (1.00 - 1.18) | 1.10 (1.01 - 1.20) | 1.07 (0.97 - 1.17) |
| Adjusted HR (95% CI) ^1^ | 1 ref | 1.04 (0.95 - 1.13) | 1.09 (1.00 - 1.18) | 1.10 (1.01 - 1.20) | 1.07 (0.98 - 1.17) |
| Cox regression analysis. All models are adjusted for age (underlying time variable) and stratified by recruitment center and age at recruitment. | | | | | |
| ^1^ Additionally adjusted for educational level (no degree, degree or unknown), smoking status (never, former, current or unknown), marital status (married, not married, unknown), diabetes (yes, no, unknown), physical activity (inactive, moderately inactive, moderately active, active, unknown), height (<170, 170–174, 175–179, ≥ 180 cm or unknown), BMI (<22.5, 22.5–24.9, 25–29.9, ≥ 30 kg/m^2^ or unknown), and total energy intake (fifths). | | | | | |
| Abbreviations: MUFAs, monounsaturated fatty acids; PUFAs, polyunsaturated fatty acids; SFAs saturated fatty acids. | | | | | |

| **Supplemental Table 7**. Multivariable-adjusted hazard ratios (95 % CI) for prostate cancer in relation to intake of fatty acids at baseline in 142,239 men in EPIC (1992-2013) by time between recruitment and diagnosis. | | | | | | | |
| --- | --- | --- | --- | --- | --- | --- | --- |
|  | **Observed intake** | | |  | **Calibrated intake** | | |
|  | **Follow-up < 5 years^1^** | **Follow-up ≥ 5 years^1^** | ***P* for het.^2^** |  | **Follow-up < 5 years^1^** | **Follow-up ≥ 5 years^1^** | ***P* for het.^2^** |
| **Total fat** | 0.95 (0.88 - 1.01) | 1.00 (0.97 - 1.03) | 0.133 |  | 0.94 (0.87 - 1.02) | 0.99 (0.96 - 1.03) | 0.199 |
| **Total SFAs** | 0.95 (0.89 - 1.02) | 1.02 (0.99 - 1.05) | 0.073 |  | 0.93 (0.85 - 1.02) | 1.01 (0.97 - 1.05) | 0.100 |
| Butyric acid (4:0) | 0.96 (0.90 - 1.03) | 1.02 (0.99 - 1.05) | 0.108 |  | 0.95 (0.88 - 1.03) | 1.02 (0.99 - 1.06) | 0.092 |
| Caproic acid (6:0) | 0.95 (0.89 - 1.02) | 1.02 (0.99 - 1.04) | 0.077 |  | 0.95 (0.88 - 1.02) | 1.02 (0.98 - 1.05) | 0.104 |
| Caprylic acid (8:0) | 0.97 (0.90 - 1.03) | 1.01 (0.98 - 1.04) | 0.214 |  | 0.96 (0.89 - 1.04) | 1.01 (0.97 - 1.04) | 0.285 |
| Capric acid (10:0) | 0.96 (0.89 - 1.04) | 1.02 (0.98 - 1.05) | 0.179 |  | 0.95 (0.87 - 1.03) | 1.02 (0.98 - 1.06) | 0.142 |
| Lauric acid (12:0) | 1.03 (0.96 - 1.10) | 1.01 (0.98 - 1.04) | 0.617 |  | 1.01 (0.93 - 1.10) | 1.00 (0.97 - 1.04) | 0.816 |
| Myristic acid (14:0) | 0.96 (0.90 - 1.03) | 1.02 (0.99 - 1.05) | 0.149 |  | 0.96 (0.88 - 1.04) | 1.01 (0.97 - 1.05) | 0.222 |
| Pentadecanoic acid (15:0) | 0.98 (0.92 - 1.05) | 0.98 (0.94 - 1.01) | 0.863 |  | 0.99 (0.92 - 1.08) | 0.97 (0.94 - 1.01) | 0.626 |
| Palmitic acid (16:0) | 0.94 (0.88 - 1.00) | 1.01 (0.99 - 1.04) | 0.036 |  | 0.91 (0.83 - 1.00) | 1.01 (0.97 - 1.05) | 0.056 |
| Margaric acid (17:0) | 0.94 (0.88 - 1.01) | 1.00 (0.97 - 1.03) | 0.108 |  | 0.93 (0.86 - 1.01) | 1.00 (0.97 - 1.04) | 0.114 |
| Stearic acid (18:0) | 0.93 (0.87 - 1.00) | 1.01 (0.98 - 1.05) | 0.042 |  | 0.90 (0.81 - 1.00) | 1.01 (0.96 - 1.05) | 0.064 |
| Arachidic acid (20:0) | 0.90 (0.81 - 0.99) | 1.00 (0.97 - 1.04) | 0.045 |  | 0.87 (0.76 - 1.00) | 1.01 (0.96 - 1.07) | 0.049 |
| Behenic acid (22:0) | 0.97 (0.89 - 1.05) | 1.00 (0.96 - 1.03) | 0.518 |  | 0.95 (0.85 - 1.07) | 1.00 (0.95 - 1.05) | 0.458 |
| 4:0-10:0 | 0.96 (0.90 - 1.03) | 1.02 (0.99 - 1.05) | 0.123 |  | 0.95 (0.88 - 1.03) | 1.02 (0.98 - 1.05) | 0.125 |
| 12:0-14:0 | 0.98 (0.92 - 1.05) | 1.01 (0.99 - 1.04) | 0.398 |  | 0.98 (0.90 - 1.06) | 1.01 (0.97 - 1.05) | 0.533 |
|  |  |  |  |  |  |  |  |
| **Total MUFAs** | 0.94 (0.85 - 1.03) | 1.00 (0.96 - 1.03) | 0.222 |  | 0.92 (0.82 - 1.04) | 0.99 (0.94 - 1.03) | 0.280 |
| Palmitoleic acid (16:1n-7c) | 0.95 (0.88 - 1.03) | 1.02 (0.98 - 1.05) | 0.129 |  | 0.90 (0.78 - 1.04) | 1.01 (0.96 - 1.08) | 0.130 |
| Oleic acid (18:1n-9c) | 0.93 (0.84 - 1.02) | 1.00 (0.96 - 1.04) | 0.153 |  | 0.91 (0.80 - 1.03) | 0.99 (0.94 - 1.04) | 0.208 |
| Eicosenoic acid (20:1n-9c) | 1.02 (0.96 - 1.07) | 1.00 (0.97 - 1.02) | 0.561 |  | 1.03 (0.96 - 1.10) | 1.00 (0.98 - 1.03) | 0.513 |
| Erucic acid (22:1n-9c) | 1.03 (0.98 - 1.09) | 1.00 (0.98 - 1.02) | 0.247 |  | 1.04 (0.97 - 1.11) | 1.00 (0.98 - 1.03) | 0.321 |
|  |  |  |  |  |  |  |  |
| **Total PUFAs** | 0.99 (0.92 - 1.07) | 0.99 (0.96 - 1.02) | 0.874 |  | 0.98 (0.89 - 1.07) | 0.98 (0.95 - 1.02) | 0.880 |
| Linoleic acid (18:2n-6c) | 0.99 (0.91 - 1.06) | 0.99 (0.96 - 1.02) | 0.965 |  | 0.96 (0.88 - 1.06) | 0.98 (0.95 - 1.02) | 0.674 |
| α-Linolenic acid (18:3n-3c) | 0.96 (0.90 - 1.03) | 0.99 (0.97 - 1.02) | 0.420 |  | 0.96 (0.88 - 1.06) | 0.99 (0.95 - 1.03) | 0.589 |
| Arachidonic acid (20:4n-6c) | 0.96 (0.89 - 1.03) | 1.00 (0.97 - 1.04) | 0.269 |  | 0.93 (0.83 - 1.04) | 1.00 (0.96 - 1.05) | 0.236 |
| Eicosapentaenoic acid (20:5n-3c) | 1.02 (0.96 - 1.08) | 1.01 (0.99 - 1.04) | 0.892 |  | 1.04 (0.96 - 1.12) | 1.01 (0.98 - 1.04) | 0.599 |
| Docosapentaenoic acid (22:5n-3c) | 1.01 (0.95 - 1.08) | 1.01 (0.98 - 1.03) | 0.847 |  | 1.01 (0.94 - 1.09) | 1.00 (0.97 - 1.03) | 0.697 |
| Docosahexaenoic acid (22:6n-3c) | 1.02 (0.96 - 1.09) | 1.01 (0.98 - 1.03) | 0.760 |  | 1.04 (0.95 - 1.13) | 1.01 (0.98 - 1.04) | 0.514 |
| **^1^** Cox regression analysis. HR (95% CI) estimated per 1 SD increase in fatty acid intake. All models are stratified by center and age at recruitment and adjusted for age (underlying time variable), educational level (no degree, degree or unknown), smoking status (never, former, current or unknown), marital status (married, not married, unknown), diabetes (yes, no, unknown), physical activity (inactive, moderately inactive, moderately active, active, unknown), height (<170, 170–174, 175–179, ≥ 180 cm or unknown), body mass index (<22.5, 22.5–24.9, 25–29.9, ≥ 30 kg/m^2^ or unknown), and total energy intake (fifths). | | | | | | | |
| ^2^ *P*-value from test for heterogeneity for the associations of fatty acid intake with risk of prostate cancer between subgroups. | | | | | | | |
| Abbreviations: MUFAs, monounsaturated fatty acids; PUFAs, polyunsaturated fatty acids; SFAs saturated fatty acids. | | | | | | | |


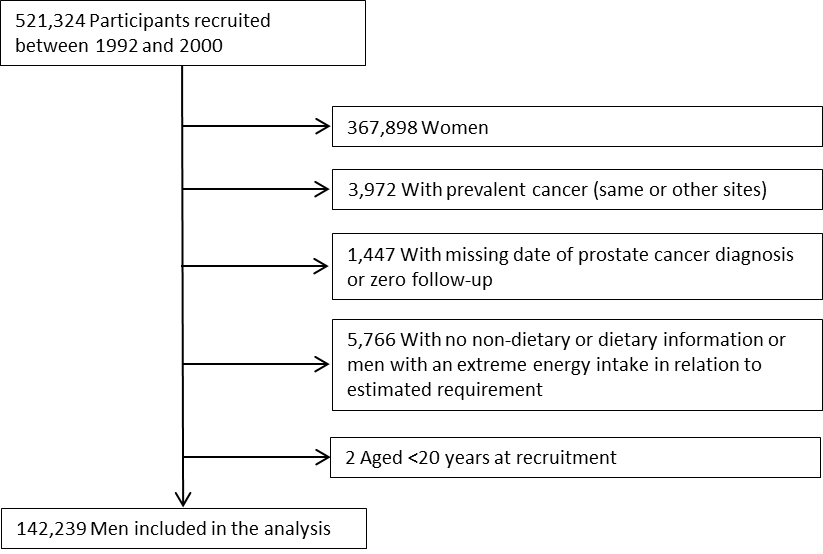


**Supplemental figure 1:** Flow chart describing the selection process among participants of the EPIC prospective study to be included in the present analyses.
